# Healthy Lifestyle, Metabolic Signature, and Risk of Cardiovascular Diseases: A Population-Based Study

**DOI:** 10.3390/nu16203553

**Published:** 2024-10-19

**Authors:** Yuhua Wang, Fei Tian, Zhengmin (Min) Qian, Shanshan Ran, Jingyi Zhang, Chongjian Wang, Lan Chen, Dashan Zheng, Michael G. Vaughn, Maya Tabet, Hualiang Lin

**Affiliations:** 1 Department of Epidemiology, School of Public Health, Sun Yat-Sen University, Guangzhou 510080, China; wangyh353@mail2.sysu.edu.cn (Y.W.); tianf8@mail2.sysu.edu.cn (F.T.); ranshsh@mail2.sysu.edu.cn (S.R.); zhangjy563@mail2.sysu.edu.cn (J.Z.); chenlan26@mail2.sysu.edu.cn (L.C.); zhengdsh5@mail2.sysu.edu.cn (D.Z.); 2 Department of Epidemiology and Biostatistics, College for Public Health & Social Justice, Saint Louis University, Saint Louis, MO 63104, USA; zhengmin.qian@slu.edu; 3 Department of Epidemiology and Biostatistics, College of Public Health, Zhengzhou University, Zhengzhou 450001, China; tjwcj2008@zzu.edu.cn; 4 School of Social Work, Saint Louis University, St. Louis, MO 63103, USA; michael.vaughn@slu.edu; 5 College of Global Population Health, University of Health Sciences and Pharmacy in St. Louis, St. Louis, MO 63110, USA; maya.tabet@uhsp.edu

**Keywords:** metabolic signature, CVD, healthy lifestyle, Mendelian randomization

## Abstract

Background: Although healthy lifestyle has been linked with a reduced risk of cardiovascular diseases (CVDs), the potential metabolic mechanism underlying this association remains unknown. Methods: We included 161,018 CVD-free participants from the UK Biobank. Elastic net regression was utilized to generate a healthy lifestyle-related metabolic signature. The Cox proportional hazards model was applied to investigate associations of lifestyle-related metabolic signature with incident CVDs, and mediation analysis was conducted to evaluate the potential mediating role of metabolic profile on the healthy lifestyle-CVD association. Mendelian randomization (MR) analysis was conducted to detect the causality. Results: During 13 years of follow-up, 17,030 participants developed incident CVDs. A healthy lifestyle-related metabolic signature comprising 123 metabolites was established, and it was inversely associated with CVDs. The hazard ratio (HR) was 0.83 (95% confidence interval [CI]: 0.81, 0.84) for CVD, 0.83 (95% CI: 0.81, 0.84) for ischemic heart disease (IHD), 0.86 (95% CI: 0.83, 0.90) for stroke, 0.86 (95% CI: 0.82, 0.89) for myocardial infarction (MI), and 0.75 (95% CI: 0.72, 0.77) for heart failure (HF) per standard deviation increase in the metabolic signature. The metabolic signature accounted for 20% of the association between healthy lifestyle score and CVD. Moreover, MR showed a potential causal association between the metabolic signature and stroke. Conclusions: Our study revealed a potential link between a healthy lifestyle, metabolic signatures, and CVD. This connection suggests that identifying an individual’s metabolic status and implementing lifestyle modifications may provide novel insights into the prevention of CVD.

## 1. Introduction

Cardiovascular disease (CVD) including ischemic heart disease (IHD), stroke, myocardial infarction (MI), and heart failure (HF), remains the leading cause of mortality, impacting around 523 million people worldwide in 2019 [1]. Most of these events are attributable to conventional life risk factors, metabolic-related diseases, and genetic factors [2]. Thus, it is necessary to reduce public health burdens via intensified management and targeted monitoring of life behaviors. Accumulated investigations have found a link between improving multiple traditional life behaviors and a lower risk of CVD, including healthy diet habits, smoking cessation, regular exercise, and normal body mass index (BMI) [3,4]. In addition, insufficient sleep has been recognized an emerging risk factor to increase the risk of CVD [5]. Despite individual lifestyle factors possibly influencing illness risk differentially, a comprehensive score could reflect combined effect and internal variability across different factors [6]. However, self-reported lifestyle evaluations may introduce measurement errors and recall biases, affecting the reliability of the results. Moreover, individuals may show different metabolic changes to similar lifestyles caused by interindividual variability.

The high-throughput metabolomic approach has offered a promising approach to discovering intermediate small molecules and metabolites, analyzing metabolic remodeling in response to external exposures, and uncovering potential mechanisms [7]. With this method, we can objectively evaluate the cumulative effect of combined lifestyle factors across the entire body and identify metabolic processes and disease pathobiology [8,9]. Previous studies have linked metabolic profiles to non-communicable diseases, such as stroke, coronary heart disease, and diabetes [10,11,12]; these outcomes are not analyzed simultaneously. Moreover, these studies found that metabolic signature could partially mediate associations of healthy lifestyle and new events. An unhealthy lifestyle can trigger immune system activation, elevate inflammatory responses and oxidative stress, and lead to insulin resistance and endothelial dysfunction, all contributing to metabolic disorders [13]. These metabolic dysfunctions play a pivotal role in the development of CVD. However, no studies have examined the extent to which metabolic profiles are associated with healthy lifestyles and CVDs.

Mendelian randomization (MR) is a method based on instrumental variable analysis that uses genetic variants to examine potential causal relationships between modifiable risk factors and disease outcomes, minimizing bias from confounding and reverse causality in observational studies [14]. By utilizing metabolomic and genetic data, MR was applied to explore the causal link between lifestyle-related metabolic profiles and CVD, offering valuable insights into identifying multiple risk factors and monitoring metabolic status as potential prevention targets for CVD.

This study aimed to identify and validate the metabolic profile associated with a healthy lifestyle, and investigate the association between the metabolic signature and risk of CVD. And we examined the mediating effects of the metabolic signature. Furthermore, we employed genome-wide association studies (GWAS) to identify the genetic connections to metabolic profiles and used MR analyses to assess the causal link between the metabolic profile and CVD.

## 2. Materials and Methods

### 2.1. Study Design and Population

The UK Biobank enrolled over 500,000 individuals aged 37 to 73 years from 2006 to 2010, collecting lifestyle and health-related information through questionnaires, verbal interviews, and physical measurements. Blood samples were also collected for genotyping. This research adhered to the principles of the Declaration of Helsinki, with written informed consent obtained from all participants.

The primary study included 180,587 participants with available data for constructing a metabolic signature. After excluding those with a history of CVD (*n* = 8413) and missing data (*n* = 11,156), the final sample consisted of 161,018 participants (Figure S1A). The genetic analysis included only individuals of European descent (*n* = 120,556).

Replication studies were conducted using follow-up data from the UK Biobank validating the primary results. Blood samples were collected between 2012 and 2014, and individuals with prevalent CVD events and missing data were excluded, leaving 10,693 participants for replication analyses (Figure S1B).

### 2.2. Construction of the Healthy Lifestyle Score

We selected five modifiable factors to create an overall healthy lifestyle score [15], including four traditional lifestyle characteristics and one emerging factor: diet, smoking status, BMI, physical activity, and sleep duration. Using a five-point scale and expressed continuously to generate an overall healthy lifestyle score, higher scores indicate a healthier lifestyle. Comprehensive definitions and classifications are provided in Table S1.

The elements of a healthy lifestyle included: a healthy dietary pattern evaluated via the frequency of consumption of fruits, vegetables, fish, and limited processed and red meat based on 24-h dietary recall questionnaires [16]; never smoking status defined as not smoking currently; a favorable BMI was considered to be less than 30 kg/m^2^; regular physical activity involving at least 150 min of moderate or mixed activity or over 75 min of intense activity per week; adequate sleep duration defined as 7 to 9 h per night [17].

### 2.3. Assessment of CVD and Follow-Up

Incident CVD, including IHD, stroke, MI, and HF were documented using medical records and mortality registry data. IHD was identified based on the 9th edition of the International Classification of Diseases (ICD) codes 410 to 414, as well as the 10th edition ICD codes I21 to I22. HF was ascertained as ICD-9 codes 428, and ICD-10 codes I11 and I13. Additionally, we employed rule-based phenotype algorithms created by the UK Biobank to detect stroke and MI cases, drawing on various sources such as Hospital Episode Statistics, Scottish Morbidity Record data, and the Patient Episode Database [18]. CVD events before recruitment and self-reported CVD events were excluded. The follow-up period spanned from registration until the earliest CVD event, death, or the last follow-up date (30 September 2021, in England; 31 July 2021, in Scotland; and 28 February 2018, in Wales), whichever came first.

### 2.4. Assessment of Covariates

Selected covariates included demographic variables, such as sex, age, ethnicity, Townsend deprivation index, educational attainment, and potential confounding factors, such as depression status. Ethnicity was categorized into white or non-white. The Townsend deprivation index measured property ownership status, assigning each participant a score based on their postal code and individual deprivation from census data [19]. Education was categorized into any school degree, college education, vocational qualifications, and other. Ascertainment of depression diagnosis was based on the definition of Davis and used self-reported depression and Patient Health Questionnaire scores [20]. Traditional risk factors included obesity, hypertension, diabetes, and dyslipidemia. Hypertension was defined via inpatient data, self-reported hypertension, use of anti-hypertensive medications, and blood pressure measurements. Diabetes was assessed via health records, self-reported diabetes, use of hypoglycemic agents, and hemoglobin A1c levels. Dyslipidemia was determined using inpatient data and the use of anti-hyperlipidemic agents.

### 2.5. Measurement of Metabolic Biomarkers

Nuclear magnetic resonance (NMR) metabolic biomarker data were generated by Nightingale Health and used for metabolomic profiling in EDTA-treated samples from around 280,000 participants in the UK Biobank [21]. A total of 16,000 participants completed the metabolome repeat assessment. The metabolites were analyzed in two periods: June 2019 to April 2020 (Phase 1) and April 2020 to June 2022 (Phase 2), using sophisticated spectrometry methods in Finland during the initial evaluation, encompassing a comprehensive profiling procedure and stringent quality assurance measures, as previously detailed [22]. The biomarkers were quantified using 170 directly measured biomarkers, comprising lipids, fatty acids, and 81 metabolite ratios, spanning multiple metabolic pathways such as the lipoprotein subclass profiling of lipid concentrations [23].

### 2.6. Genotyping and Imputation

Participants were genotyped using the UK BiLEVE or UK Biobank Axiom arrays, with imputation based on UK10K and 1000 Genomes data [24]. Quality control excluded low-quality samples, and subsequent analysis included only white British individuals. After standardization, 251 metabolic markers were qualified for our analysis. We excluded genetic variants if their minor allele frequency was over 0.5% and their info score was under 0.3, or if they failed Hardy–Weinberg equilibrium tests with a *p*-value less than 1 × 10^−6^.

### 2.7. Statistical Analysis

Multivariable linear regression was used to analyze the associations between selected metabolic biomarkers and individual lifestyle choice and Bonferroni corrections were applied to ensure statistical significance.

Firstly, using baseline metabolomics, we generated a metabolic signature linked to healthy lifestyle and tested it using initial repeat metabolites data. To standardize the 251 metabolites, we first performed a log transformation and then applied z-scores to ensure a consistent scale. Next, to identify the metabolites related to healthy lifestyle, we employed elastic net regression approach. This method combines the strengths of two techniques, lasso and ridge regression, to address multicollinearity and prevent overfitting, which can otherwise reduce the reliability of the model, by using an alpha value of 0.5. By using elastic net regression, we could focus on the most important metabolites while maintaining a stable model. In this analysis, we examined how the total healthy lifestyle score was related to the 251 metabolites to create a metabolic signature that reflected a healthy lifestyle. To ensure accuracy, we used a 10-fold cross-validation process, where the data were split into 10 equal parts. In each round, the model was trained on 9 parts and tested on the remaining part. This process was repeated 10 times to obtain a reliable estimate of the model’s performance. Through this process, we identified the best value for a parameter called lambda, which helped control the model’s complexity. By selecting the optimal lambda value (lambda value = 0.0007), we minimized prediction errors, as measured via the mean squared error, and ensured the model’s accuracy was within 1 standard deviation of the minimum error (RMSE = 0.95) [25]. The metabolic signature was calculated by combining the metabolite values, with each one weighted according to its importance determined via the elastic net regression. Finally, we applied this trained model to separate testing datasets to calculate the metabolic signature and confirm our results via replication studies. The Pearson correlation method was employed to investigate the relationships among the 251 metabolites and to further explore the association between the metabolic profile and healthy lifestyle score. To observe patterns in the metabolic signature, we recategorized it according to three tertiles: unfavorable (tertile 1), moderate (tertile 2), and favorable (tertile 3).

Secondly, we calculated hazard ratios (HRs) and 95% confidence intervals (CIs) to examine the relationships between a healthy lifestyle score, metabolic profile, and incident CVD using Cox proportional hazards regression models. The Schoenfeld residuals were employed to validate the assumption of proportional hazards. To minimize confounding bias, we established three models. Model 1 included sex and age. Model 2 added ethnicity, Townsend deprivation index, education, and prevalent depression. Model 3 further adjusted for the healthy lifestyle score and metabolic signature. Notably, the replication sample were conducted to perform an internal validation. We also analyzed the relationship between metabolites, metabolic pathways, lifestyle factors, and CVD events. To assess the possible non-linear relationships of healthy lifestyle and metabolic signature on CVD, we employed restricted cubic spline regression with three knots.

Thirdly, mediation analyses were conducted to determine whether the association between the overall healthy lifestyle score and the risk of CVD could be partially mediated through metabolic factors (mediator). To explore the underlying biological mechanisms and clarify the mediating effects, we assessed the extent to which metabolic profiles accounted for the relationship between lifestyle and CVD.

Based on the previous estimation [26], the proportion of the total effect attributable to a mediator was determined by dividing the mediation effect of the metabolic profile by the total effect of a healthy lifestyle on CVD incidence. Additionally, we explored different metabolic pathways contributing to these relationships and quantified the contribution of each metabolic biomarker. These analyses were performed with the R package ‘mediation’ using 100 simulations, providing proportions and their corresponding 95% confidence intervals. These models were adjusted according to the specifications of Model 3.

Fourthly, we applied GWAS to identify novel links between chromosomal loci and disease as well as to detect significant single nucleotide polymorphisms (SNPs) in unknown genes. We employed linear regression analysis to evaluate the association between phenotypes and metabolic profiles using an additive genetic model. The model incorporates individual genetic variations, age, sex, healthy lifestyle, and the first ten principal components. We utilized the GWAS results of metabolic signatures identified in the primary study, along with summary statistics for CVD from various GWAS datasets, to perform two-sample MR analyses in line with three core assumptions previously outlined [27]. This method was employed to test the potential causal relationships between the metabolic profile and four diseases risk. The genetic instruments were selected based on a *p* value below 1 × 10^−6^, and these SNPs were filtered for independence using a clumping algorithm with an r^2^ threshold of 0.01 and a distance window of 10,000 kb. We conducted these meta-analyses for different diseases to derive overall estimates from multiple public datasets and determine whether heterogeneity existed. Furthermore, we evaluated the reliability of our findings via weighted median, MR-Egger, and weighted model analyses.

Sensitivity analyses were performed, such as stratifying by sex and age (<60 years or ≥60 years), excluding new CVD cases within the initial 2 years of follow-up, excluding existing cancer cases, further adjusting for traditional risk factors, medication with cardioprotective effects, and imputating missing lifestyle information. All statistical analyses were conducted using two-sided tests with a significance level of *p* < 0.05. All data processing was performed in R, version 4.2.2.

## 3. Results

### 3.1. Characteristics of the Study Participants

Over a median follow-up of 13 years, we identified 17,030 incident CVD cases, of which 12,599 were IHD, 2898 were stroke, 2391 were MI, and 4281 were HF. And the follow-up period in this study ranged from 1 to 16 years. Table 1 displays the baseline characteristics of the study participants based on incident CVD status. Of 161,018 participants, the mean age was 56.0 years, and 46.5% of participants were men. The replication study included 10,693 participants, with an average age of 56.6 years, of whom 49.0% were men, showing a similar pattern to the primary study (Table S2). Those with CVD were more likely to be older, male, and obese. They were also more likely to be physically inactive, less educated, less inclined to adhere to a healthy lifestyle, and have a higher burden of traditional CVD risk factors.

### 3.2. Identification of the Metabolic Signature Related to Healthy Lifestyle

**Figure 1:**
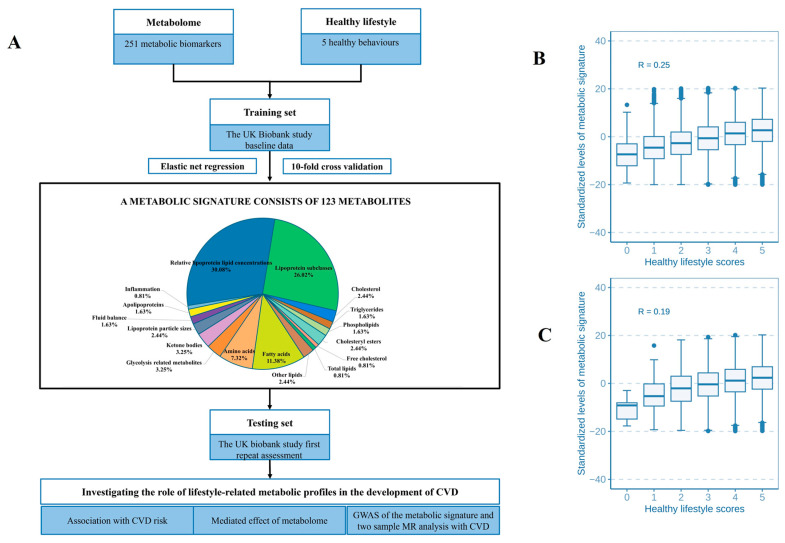
Flowchart of study design and associations between the metabolic signature and healthy lifestyle score. (**A**) The training and testing procedures of a metabolic signature for the healthy lifestyle. The correlations between healthy lifestyle scores and the metabolic signature in the primary and replication study are shown in (**B**,**C**).

**Figure 2:**
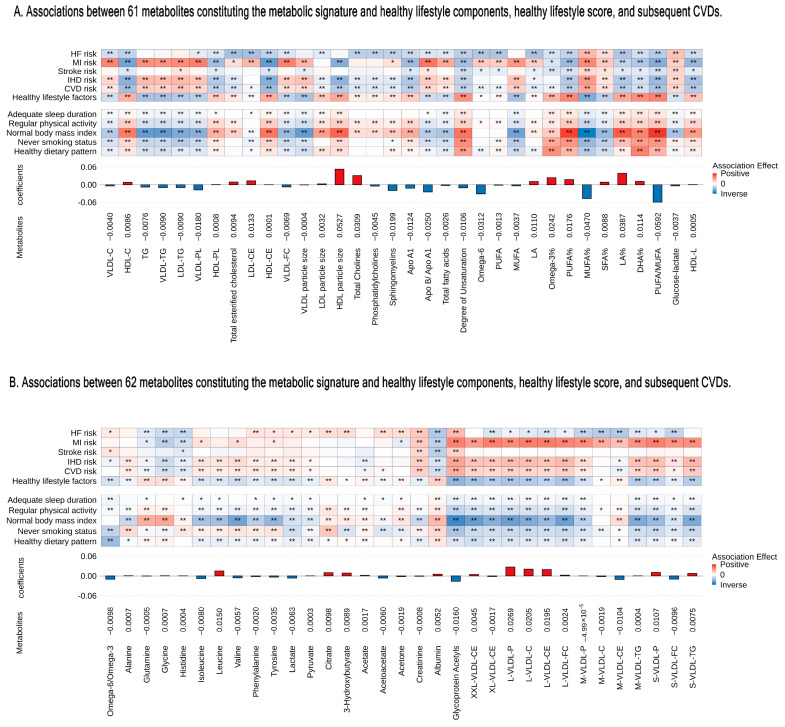
Associations between 123 metabolites constituting the metabolic signature and healthy lifestyle components, healthy lifestyle score, and subsequent CVDs. (**A**) For 61 metabolites. (**B**) For 62 metabolites. From bottom to top are the metabolites’ coefficients (weights) of the signature and associations with healthy lifestyle component, healthy lifestyle, and CVDs. The coefficients for healthy lifestyle components and the overall healthy lifestyle score represent a one-unit increase per component’s score. Coefficients for CVD risk correspond to per SD increase in metabolites. Colors depict the direction of associations (positive-red and inverse-blue) and their magnitudes (darker shades indicate larger magnitudes); asterisks denote significance levels (* *p* < 0.05 and ** *p* < Bonferroni-adjusted 0.05). We used Bonferroni correction for 123 metabolites for the healthy lifestyle score and CVD risk and 123 metabolites × 5 healthy lifestyle components). C indicates cholesterol; CE, cholesteryl ester; FC, free cholesterol; LDL, low-density lipoprotein; M, medium; PL, phospholipid; S, small; VLDL, very LDL; XL, very large; XS, very small; XXL, extra large; SD, standard deviation; CVD, cardiovascular disease; IHD, ischemic heart disease; MI, myocardial infarction; and HF, heart failure.

The correlation matrix showed a high correlation among 251 metabolites (Figure S2). The analysis pinpointed 123 metabolites to identify a metabolic profile, demonstrating a correlation with healthy lifestyle scores (UK Biobank baseline: R = 0.25, *p* < 2.2 × 10^−16^; follow-up: R = 0.20, *p* < 2.2 × 10^−16^) (Figure 1B,C). The metabolites traversed various metabolic pathways, including lipoprotein lipid concentrations, lipoprotein subclasses, fatty acids, and 14 other pathways (Figure 1A and Table S3). Of the 120 associated metabolites, 59 showed a positive correlation with a healthy lifestyle score (Figure 2A,B). Significantly, beneficial elements influencing the metabolic profile were the polyunsaturated fatty acids to monounsaturated fatty acids ratio (PUFA/MUFA), polyunsaturated fatty acids to total fatty acids percentage (PUFA%), and docosahexaenoic acid to total fatty acids percentage (DHA%). On the other hand, monounsaturated fatty acids to total fatty acids percentage (MUFA%) showed the most negative correlation (Figure 2A,B).

Different lifestyle factors were associated with distinct subsets of selected metabolites, with stronger and more consistent associations observed for dietary pattern, smoking status, and BMI, as illustrated in Figure 2A,B. For example, a healthy BMI showed a positive correlation with PUFA/MUFA, PUFA%, and linoleic acid to total fatty acids percentage (LA%). Additionally, we identified 13 metabolic pathways that were positively linked to the healthy lifestyle score (Figure S3). Notably, the metabolic signature varied significantly even with identical healthy lifestyle scores, due to variations in the components of the score and other factors (Figure 1B,C).

### 3.3. Associations of Healthy Lifestyle with the Risk of CVD

Following a healthy lifestyle was linked to a reduced risk of all outcomes in the fully adjusted models of the main study. The HRs of CVD, IHD, stroke, MI, and HF were 0.85 (95% CI: 0.84, 0.86), 0.85 (95% CI: 0.84, 0.87), 0.89 (95% CI: 0.86, 0.92), 0.84 (95% CI: 0.81, 0.87), and 0.78 (95% CI: 0.76, 0.80) per SD increase in the healthy lifestyle score, respectively (Table 2). In addition, we found that specific healthy lifestyle behaviors reduced the likelihood of developing CVD (Table S4). The link between a healthy lifestyle and other illnesses remained stable except in the replication study on stroke and MI (Table S5).

### 3.4. Associations of the Metabolites, Metabolic Signature, and Metabolic Pathways with the Risk of CVD

Our research identified 108 metabolites linked to CVD risk, including phospholipids to total lipids in a very small VLDL percentage (XS-VLDL-PL%), MUFA%, cholesteryl esters in HDL (HDL-CE), and a concentration of large HDL particles (L-HDL-P) (Figure 2A,B). Furthermore, we observed a consistent protective effect of the metabolic signature after full adjustment. The HRs per SD increases were as follows: for CVD, HR: 0.83 (95% CI: 0.81, 0.84); for IHD, HR: 0.83 (95% CI: 0.81, 0.84); for stroke, HR: 0.86 (95% CI: 0.83, 0.90); for MI, HR: 0.86 (95% CI: 0.82, 0.89); and for HF, HR: 0.75 (95% CI: 0.72, 0.77). In the replication study, we observed the protective effects of the metabolic signature, with a reduction in CVD risk of 0.82 (0.76, 0.88). These associations remained statistically significant, except for stroke. Associations with the risk of CVD were attenuated among participants with favorable metabolic signatures [HR: 0.67 (95% CI: 0.65, 0.70)] (Table S6). Our findings indicate that a favorable metabolic profile might be associated with a lower risk of various illnesses, supported by a dose–response trend (Figure S4).

**Figure 3:**
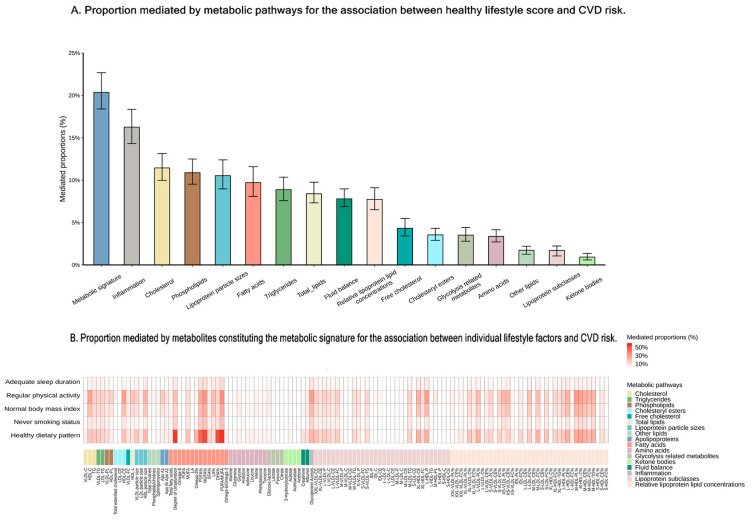
Proportions mediated by metabolites or the metabolic signature for the association between healthy lifestyle and CVDs risk. (**A**) Proportion mediated by metabolic pathways for the association between healthy lifestyle score and CVDs risk. (**B**) Proportion mediated by metabolites constituting the metabolic signature for the association between individual lifestyle factors and CVD risk. Statistical significance was set at *p*  <  0.05. CVD indicates cardiovascular disease; IHD, ischemic heart disease; MI, myocardial infarction; and HF, heart failure.

To elucidate metabolic mechanisms, we assessed the associations between metabolic pathways and CVD. All metabolic pathways were linked to CVD risk, with five to fifteen pathways associated with specific disease (Figure S3). The consistent metabolic pathways were identified across each disease process, particularly those involving lipids, fatty acids, and lipoprotein subclasses (Figure S3). Our analysis showed that 20% of the beneficial effect of a healthy lifestyle on CVD was mediated by the metabolic signature, with 16 pathways identified as significant contributors to this mediation effect, including inflammation, cholesterol, and phospholipids as the top three pathways (Figure 3A and Figure S5A). Specifically, we observed that the metabolic profile accounted for 15.6% to 21.6% of the association between a healthy lifestyle and the incidence of four distinct diseases (Figure S5B). Additionally, we estimated the effect of individual metabolites linked to specific healthy lifestyle components in mediating CVD risk, with significant contributions particularly from fatty acids (Figure 3B). Notably, the reduced CVD risk associated with a healthy dietary pattern is likely significantly mediated by PUFA% and MUFA%.

**Figure 4:**
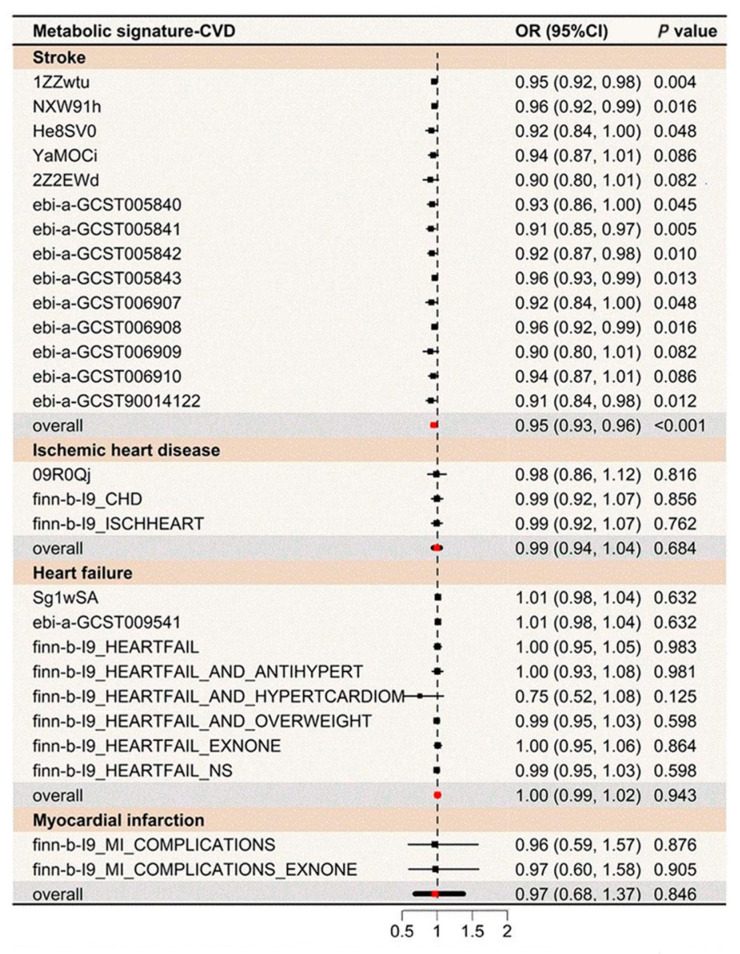
MR analyses for the association between the metabolic signature and CVDs risk. Fourteen genetic variants linked to the metabolic signature at *p* < 1 × 10^−6^ were employed as the instrumental variable in the mode-based estimation (MBE) MR analyses following Hartwig’s method. OR indicates odds ratio; CI, confidence interval; MR, Mendelian randomization; CVD, cardiovascular disease; IHD, ischemic heart disease; MI, myocardial infarction; and HF, heart failure.

To evaluate the influence of genetics on the metabolic profile associated with healthy lifestyle, GWAS identified nine lead SNPs across eight genomic risk loci (Table S7). MR meta-analyses indicated that the genetic component of the metabolic profile was associated with a lower stroke risk (OR: 0.95, 95% CI: 0.93, 0.96), but evidence for other diseases was limited (Figure 4). Besides the IEU global GWAS project, three additional GWAS datasets were analyzed to confirm the outcomes (Table S8). Two-sample MR analyses provided estimates for the metabolic signature and CVD, with consistent results in sensitivity analyses (Tables S9 and S10).

### 3.5. Sensitivity Analysis

The sensitivity results remained consistent across age and sex stratifications (Figure S6). Additional analyses, excluding early events, cancer cases, and further traditional risk factor adjustments, showed similar associations between healthy lifestyle, metabolic profiles, and CVD (Tables S11–S13). Moreover, adjusting for cardioprotective medications did not affect our findings (Table S14). Interpolating missing lifestyle data did not change the results (Table S15).

## 4. Discussion

### 4.1. Overview of Results

Using a large prospective cohort, we identified 123 metabolites to develop and validate a metabolic signature that captures the long-term effects of various lifestyle choices. The metabolic signature was linked to a reduced risk of CVD and may mediate the beneficial effects of lifestyle interventions on incident CVD. In addition, MR results indicated a potential causal relationship between the metabolic profile and stroke. Our findings offer novel insights into integrating lifestyle recommendations and metabolite screening into the optimal treatment and risk stratification of CVD.

### 4.2. Comparison with Previous Literature

A substantial body of research has established the reliability and validity of self-reported lifestyle variations, revealing strong correlations with metabolism, including overall metabolic dynamics and specific metabolites [28,29,30,31]. By identifying 123 metabolic biomarkers linked to five lifestyle choices, we developed a metabolic profile that offers protection from CVD. Our findings contribute to the existing literature and suggest that a metabolic profile may be a valuable tool for assessing healthy lifestyle in relation to a spectrum of disease risks. As an example, in the PREDIMED study of 1833 participants, 58 metabolites were selected to identify a metabolic signature associated with an 8-scale lifestyle score, which was found to prevent new cases of CVD and diabetes [32]. Additionally, research from the US NHANES and the UK Biobank further supports the strong association between maintaining a healthy lifestyle and a reduced incidence of CVD [33]. A matched case–control and cohort study identified 161 metabolic indicators within a metabolic signature associated with an overall healthy lifestyle score, which could help lower the risk of CHD [34]. However, no studies have simultaneously analyzed the four vascular diseases (including MI, HF, IHD, and stroke) or any one of them to examine how a metabolic signature might be influenced by lifestyle choices.

The incorporation of lipid marker panels (HDL-C, LDL-C, and triglyceride) into the SCORE risk chart for screening has been recommended, particularly for high-risk groups with a high genetic predisposition or hyperlipidemia, to optimize CVD risk assessment and refine risk stratification [35]. Prior investigations have established a significant link between glycoprotein acetyls, apolipoprotein B, lipids, and the risk of CVD in individuals with psoriasis and those with diabetes [36,37]. Although these metabolites were extracted using a standardized platform with high accuracy and precision, the analyses focused on individuals with existing diseases rather than a healthy population. Furthermore, a recent study identified and validated a metabolic profile linked to the Mediterranean diet, emphasizing its potential as a reliable risk predictor for CVD [22]. Our findings align with this evidence, highlighting the important role of lifestyle in lipid and fatty acid metabolism in the development of CVD. Our GWAS results revealed that the metabolic signature was significantly affected by the gene loci including DOCK7 and the Interleukin-6 receptor (IL6R). In large-scale cross-sectional analyses, the DOCK7 variant has been identified as a genetic determinant of blood lipid levels, particularly in increasing triglyceride levels, which contributes to a higher risk of coronary artery disease [38]. IL6R, an inflammatory cytokine, modulates biomarkers of inflammation, such as interleukin 6, C-reactive protein, fibrinogen, hemoglobin, and albumin [39]. Additionally, we identified a comparable potential causal relationship between the metabolic signature and stroke. However, a small portion of the MR analyses showed non-significant associations between the metabolic signature and CVD. Further studies are needed to incorporate a broader range of modifiable lifestyle factors when developing accurate metabolic profiles for effective CVD prevention. Another notable finding is the low performance of the metabolic signature. Previous epidemiological studies have demonstrated strong correlations between healthy lifestyle scores and metabolic signatures [8,40]. For instance, a study of 87,258 individuals in the UK Biobank found that a metabolic signature comprising 81 metabolic biomarkers was correlated with the healthy lifestyle score (R = 0.45). In contrast, our analysis showed a weaker correlation. A potential explanation could be differences in the lifestyle components and disease susceptibility across studies. Future research should incorporate additional lifestyle factors to develop a more comprehensive metabolic signature that better reflects the metabolic response to each lifestyle component. The underlying mechanisms linking lifestyle-related metabolites to incident CVD have been postulated, but remain insufficiently understood. Our study found that the metabolic signature mediates the protective association between maintaining a healthy lifestyle and the risk of CVD. Specifically, the metabolic profile accounted for 21.6% of the association with stroke, 20.7% with IHD, 20.6% with HF, and 15.6% with MI. Among the 17 metabolic pathways analyzed, the most significant contributors to this mediation were inflammation, cholesterol, and phospholipids. Lifestyle improvements have been proven to maintain metabolic balance, thereby supporting cardiovascular health. For example, routine exercise exerts beneficial effects that extend beyond the favorable modulation of serum lipid profiles and blood pressure. It enhances cardioprotection by mitigating oxidative stress damage, increasing ATP-sensitive potassium channels, and maintaining mitochondrial resilience [41]. Furthermore, exercise promotes plaque stability by boosting collagen and elastic fiber content, which counteracts the effects of disease and aging on muscles [42]. Additionally, exercise training reduces inflammation, as evidenced by decreased CRP concentrations in otherwise healthy adults, helping to protect arteries from atherosclerosis and stabilize existing plaques [43]. Moreover, a lower omega-6/omega-3 ratio and increased PUFAs have been associated with reduced vascular incidents [44]. Given these findings, we hypothesize that metabolism plays a critical role in the relationship between lifestyle factors and the incidence of CVD.

Our study was the first to identify 123 metabolites to establish and validate a metabolic profile associated with a healthy lifestyle. This profile reflects comprehensive changes in metabolic patterns linked to various lifestyle choices and the body’s metabolic response to lifestyle improvements. These findings underscore the potential for clinical treatment and precision strategies utilizing surrogate markers in the management and prevention of vascular diseases. By using the metabolic signature to model the variation in lifestyle behaviors, we provided a more objective and accurate evaluation of CVD susceptibility compared to self-reported lifestyle data. Additionally, we observed a proportional relationship between the metabolic profile and a reduced risk of four diseases, indicating that key metabolic pathways and inflammatory markers likely play a crucial role in the pathogenesis of CVD. They also contribute to a deeper understanding of the role of biosynthetic pathways in maintaining metabolic equilibrium. Furthermore, our results indicate that targeted lifestyle modifications and the use of metabolic biomarkers could be pivotal in mitigating the detrimental effects on CVD health.

### 4.3. Strengths and Limitations

This study was conducted to investigate the association between healthy lifestyle-related metabolic profiles and the risk of CVD. The strengths include a large cohort size, the availability of detailed information on lifestyle, and the use of high-dimensional data-based elastic net regression. However, it is important to acknowledge several limitations. Firstly, lifestyle data were self-reported on five traits at baseline; participants might change their lifestyle over the course of the follow-up, potentially obscuring the true link between lifestyle behaviors and CVD risk. Further studies should include a broader range of traits to strengthen the analysis. Secondly, the overall healthy lifestyle score was unweighted, which may not accurately reflect the independent contributions of different behaviors. Thirdly, multicollinearity may mislead interpretations of the results. Fourthly, the main analysis was not replicated in an external cohort, possibly affecting the consistency of findings. Fifthly, only a proportion of metabolites were identified using the Nightingale Health platform; future studies could benefit from utilizing multiple platforms to identify previously unrecognized metabolites and more accurately capture metabolic dynamics. Finally, the study’s participants were from European backgrounds, restricting the applicability of the results to other groups.

### 4.4. Implications for Public Health

The metabolic signature reflects homeostasis by integrating the interrelated effects of genetic predisposition, chronic conditions, and lifestyle practices [22]. Via high-throughput metabolomic analysis, we identified 123 metabolic biomarkers to develop a metabolic signature that measures the body’s metabolic response to lifestyle behaviors, independent of self-reported lifestyle information, for predicting CVD risk. This approach enhances traditional methods by utilizing metabolites to better capture lifestyle influences and guide prevention strategies for reducing CVD risk. Furthermore, we uncovered a potential causal relationship between the metabolic signature and stroke risk, underscoring the importance of metabolic balance and lifestyle modification in risk management and clinical practice for stroke. Additionally, we found that the metabolic signature may mediate the associations between a healthy lifestyle and CVD risk, suggesting underlying biological pathways in the development of CVD.

## 5. Conclusions

Using a prospective cohort of 161,018 participants over 13 years, we identified 123 metabolites to develop and validate a metabolic signature and observed an inverse between the metabolic signature and CVD risk. Furthermore, we identified a set of plasma metabolites that facilitate the early identification of high-risk individuals, thereby complementing the improvement and management of traditional risk factors.

## Figures and Tables

**Table 1:** Baseline characteristics of participants by CVD incidence.

Characteristics	Overall (*n* = 161,018)	Incident CVD (*n* = 17,030)	No CVD (*n* = 143,988)	*p*-Value *
Age, years	56.00 (8.02)	60.23 (6.88)	55.50 (8.00)	<0.001
Male	74,885 (46.51)	10,469 (61.47)	64,416 (44.74)	<0.001
Townsend deprivation index	−1.66 (2.89)	−1.41 (3.03)	−1.69 (2.87)	<0.001
Education level				<0.001
Any school degree	62,998 (39.12)	6072 (35.65)	56,926 (39.54)	
College education	58,263 (36.18)	4727 (27.76)	53,536 (37.18)	
Other education	29,632 (18.40)	4794 (28.15)	24,838 (17.25)	
Vocational qualification	10,125 (6.29)	1437 (8.44)	8688 (6.03)	
Metabolic signature score	0.62 (7.16)	−1.62 (7.37)	0.88 (7.09)	<0.001
Metabolic signature level				<0.001
Unfavorable	49,589 (30.80)	7324 (43.01)	42,265 (29.35)	
Moderate	54,716 (33.98)	5451 (32.01)	49,265 (34.21)	
Favorable	56,713 (35.22)	4255 (24.99)	52,458 (36.43)	
Healthy lifestyle score	3.76 (1.02)	3.55 (1.09)	3.78 (1.01)	<0.001
Healthy lifestyle components				
Healthy diet	131,573 (81.71)	13,527 (81.15)	118,046 (83.14)	<0.001
No current smokers	145,564 (90.40)	14,811 (86.97)	130,753 (90.81)	<0.001
Body mass index < 30 kg/m^2^	123,265 (76.75)	11,524 (67.95)	111,741 (77.79)	<0.001
Regular physical activity	85,429 (53.06)	8662 (50.86)	76,767 (53.31)	<0.001
Adequate sleep duration	118,796 (75.81)	11,875 (72.30)	106,921 (76.22)	<0.001
Prevalent depression status	5161 (3.21)	531 (3.12)	4630 (3.22)	0.509
Traditional risk factors				
Obesity	37,345 (23.25)	5436 (32.05)	31,909 (22.21)	<0.001
Hypertension	109,887 (68.25)	14,262 (83.75)	95,625 (66.41)	<0.001
Diabetes	6589 (4.09)	1597 (9.38)	4992 (3.47)	<0.001
Dyslipidemia	20,823 (12.93)	4422 (25.97)	16,401 (11.39)	<0.001

* *p* values were calculated via *t* test, χ^2^ test, or Fisher’s exact test, as appropriate. Values are mean ± SD, *n* (%), median (IQR), or *n*/*n* (%). SD indicates standard deviation; and CVD, cardiovascular disease.

**Table 2:** Associations between the healthy lifestyle score and the metabolic signature and risk of incident CVDs.

Analysis Model	No. of Events	Healthy Lifestyle Score *		Metabolic Signature ^†^	
		HR (95% CI)	*p*-Value	HR (95% CI)	*p*-Value
CVD					
Age- and sex-adjusted model	17,030	0.79 (0.78, 0.80)	<0.001	0.78 (0.76, 0.79)	<0.001
Multivariable-adjusted model ^‡^	17,030	0.81 (0.80, 0.83)	<0.001	0.79 (0.78, 0.81)	<0.001
Multivariable-adjusted + mutual adjustment ^§^	17,030	0.85 (0.84, 0.86)	<0.001	0.83 (0.81, 0.84)	<0.001
IHD					
Age- and sex-adjusted model	12,599	0.80 (0.78, 0.81)	<0.001	0.78 (0.76, 0.79)	<0.001
Multivariable-adjusted model ^‡^	12,599	0.82 (0.81, 0.83)	<0.001	0.80 (0.78, 0.81)	<0.001
Multivariable-adjusted + mutual adjustment ^§^	12,599	0.85 (0.84, 0.87)	<0.001	0.83 (0.81, 0.84)	<0.001
Stroke					
Age- and sex-adjusted model	2898	0.84 (0.81, 0.87)	<0.001	0.82 (0.79, 0.85)	<0.001
Multivariable-adjusted model ^‡^	2898	0.86 (0.83, 0.89)	<0.001	0.84 (0.81, 0.87)	<0.001
Multivariable-adjusted + mutual adjustment ^§^	2898	0.89 (0.86, 0.92)	<0.001	0.86 (0.83, 0.90)	<0.001
MI					
Age- and sex-adjusted model	2391	0.79 (0.76, 0.82)	<0.001	0.80 (0.76, 0.83)	<0.001
Multivariable-adjusted model ^‡^	2391	0.81 (0.78, 0.84)	<0.001	0.82 (0.78, 0.85)	<0.001
Multivariable-adjusted + mutual adjustment ^§^	2391	0.84 (0.81, 0.87)	<0.001	0.86 (0.82, 0.89)	<0.001
HF					
Age- and sex-adjusted model	4281	0.70 (0.68, 0.72)	<0.001	0.68 (0.66, 0.70)	<0.001
Multivariable-adjusted model ^‡^	4281	0.73 (0.71, 0.75)	<0.001	0.70 (0.68, 0.72)	<0.001
Multivariable-adjusted + mutual adjustment ^§^	4281	0.78 (0.76, 0.80)	<0.001	0.75 (0.72, 0.77)	<0.001

* HR and 95% CI of CVDs risk per SD increment in healthy lifestyle scores. ^†^ HR and 95% CI of CVDs risk per SD increment in metabolic signature. ^‡^ Based on an age- and sex-adjusted model, further adjusted for ethnicity, Townsend deprivation index, education, and depression status. ^§^ Included both healthy lifestyle score and the metabolic signature simultaneously in the multivariable-adjusted model to examine association independence. HR indicates hazard ratio; CI, confidence interval; SD, standard deviation; CVD, cardiovascular disease; IHD, ischemic heart disease; MI, myocardial infarction; and HF, heart failure.

## Data Availability

The datasets generated and analyzed during the current study are available upon reasonable request to the Access Management System (AMS) via the UK Biobank website (https://www.ukbiobank.ac.uk/enable-your-research/apply-for-access, accessed on 25 March 2024).

## Supplementary Materials

The following supporting information can be downloaded at: https://www.mdpi.com/article/10.3390/nu16203553/s1, Table S1. The detailed definition of lifestyle factors included in healthy lifestyle scores. Table S2. Baseline characteristics of participants by CVD incidence in the replication study. Table S3. Metabolite information included in the metabolic signature. Table S4. Associations of specific healthy lifestyle factors with CVDs in the primary study. Table S5. Associations between healthy lifestyle, metabolic signature, and risk of incident CVDs in the replication study. Table S6. Associations of metabolic signature levels with risk of incident CVDs. Table S7. Summary of 8 independent loci associated with metabolic signature. Table S8. The basic characteristics of GWAS summary statistic datasets for the metabolic signature and CVDs. Table S9. MR results for the effect of metabolic signatures on CVDs risk. Table S10. MR sensitivity analyses investigating the effect of the metabolic signature on CVDs. Table S11. Sensitivity analyses for associations of metabolic signature and healthy lifestyle with CVDs risk by excluding new cases within 2 years. Table S12. Sensitivity analyses for associations of metabolic signature and healthy lifestyle with CVDs risk adjustment for prevalent cancer diseases. Table S13. Sensitivity analyses for associations of metabolic signature and healthy lifestyle with CVDs risk adjusted for traditional risk factors. Table S14. Sensitivity analyses for associations of metabolic signature and healthy lifestyle with CVDs risk adjusted for medications. Table S15. Sensitivity analyses for associations of metabolic signature and healthy lifestyle with CVDs risk with imputation of incomplete lifestyle factors. Figure S1. Flowchart of study participant selection. Figure S2. Correlation matrix for the 251 metabolites. Figure S3. Associations of the 17 metabolic pathways constituting the metabolic signature and healthy lifestyle components, healthy lifestyle score, and subsequent CVDs. Figure S4. Dose–response relationship between healthy lifestyle, metabolic signature, and CVDs risk using restricted cubic spline analysis. Figure S5. Mediation analyses of the association of healthy lifestyle with different diseases by the metabolic signature. Figure S6. Sensitivity analyses for associations of metabolic signature and healthy lifestyle with CVDs risk stratified by age and sex.
