# Supplementary material for: Healthy Lifestyle, Metabolic Signature, and Risk of Cardiovascular Diseases: A Population-Based Study
Source: Nutrients. 2024 Oct 19;16(20):3553. doi: 10.3390/nu16203553 (PMC11510148; doi:10.3390/nu16203553)
Supplement: Supplementary file 1 [file nutrients-16-03553-s001.zip › Supplementary materials.pdf]

Table S1. The detailed definition of lifestyle factors included in healthy lifestyle scores

Lifestyle factors	Source and definition	UK Biobank data fields
**Dietary pattern**	UK Biobank Food Frequency Questionnaire at baseline<br>A healthy diet was defined as meeting at least two of the dietary items.<br>1. Fruit and vegetable: ≥4.5 servings/day<br>[Three tablespoons of vegetables were considered one serving]<br>2. Fish: ≥2 servings/week<br>3. Processed meat≤ 2 times/week &<br>red meat≤5 times/week	1289: Cooked vegetables intake<br>1299: Salad / raw vegetable intake<br>1309: Fresh fruit intake<br>1329: Oily fish intake<br>1339: Non-oily fish intake<br>1349: Processed meat intake<br>1369: Beef intake<br>1379: Lamb/mutton intake<br>1389: Pork intake
**Smoking status**	UK Biobank Touchscreen questionnaire at baseline	1239: Current tobacco smoking
**Body mass index**	BMI is constructed from height and weight measured during the initial Assessment Centre visit	21001: Body mass index (BMI)
**Physical activities**	UK Biobank Touchscreen questionnaire at baseline;<br>Regular physical activity level was defined as meeting at each of the dietary items.<br>1.≥150 min/week moderate activity<br>2.≥75 min/week vigorous activity<br>3.≥150 min/week moderate and vigorous activity	884: Number of days/week of moderate physical activity 10+ minutes<br>894: Duration of moderate activity<br>904: Number of days/week of vigorous physical activity 10+ minutes<br>914: Duration of vigorous activity
**Sleep duration**	UK Biobank Touchscreen questionnaire at baseline;<br>Adequate sleep duration was defined as meeting 7-9 hours of sleep duration each day.	1160: hours sleep in every 24 hours (include naps)

Table S2. Baseline characteristics of participants by CVD incidence in the replication study

	Overall (n=10,693)	Incident CVD (n=815)	No CVD (n=9,878)
**Age, years**	56.58 (7.40)	60.68 (6.22)	56.24 (7.39)
**Male**	5,241 (49.01)	524 (64.29)	4,717 (47.75)
**Townsend deprivation index**	-2.14 (2.60)	-2.23 (2.59)	-2.14 (2.61)
**Education level**			
Any school degree	3,744 (35.01)	264 (32.39)	3,480 (35.23)
College education	5,113 (47.82)	330 (40.49)	4,783 (48.42)
Other education	1,233 (11.53)	152 (18.65)	1,081 (10.94)
Vocational qualification	603 (5.64)	69 (8.47)	534 (5.41)
**Metabolic signature score**	0.71 (7.12)	-1.67 (7.30)	0.91 (7.07)
**Metabolic signature level**			
Unfavorable	3,288 (30.75)	337 (41.35)	2,951 (29.87)
Moderate	3,635 (34.00)	294 (36.07)	3,341 (33.82)
Favorable	3,770 (35.26)	184 (22.58)	3,586 (36.30)
**Healthy lifestyle score**	3.89 (0.96)	3.79 (1.01)	3.90 (0.95)
**Healthy lifestyle components**			
Healthy diet	8,899 (83.84)	660 (81.89)	8,239 (84.00)
No current smokers	10,061 (94.09)	759 (93.13)	9,302 (94.17)
Body mass index <30 kg/m^2	8,710 (81.58)	622 (76.51)	8,088 (82.00)
Regular physical activity	5,697 (53.28)	431 (52.88)	5,266 (53.31)
Adequate sleep duration	8,219 (78.41)	619 (78.35)	7,600 (78.42)
**Prevalent depression status**	524 (4.90)	37 (4.54)	487 (4.93)
**Traditional risk factors**			
Obesity	1,967 (18.40)	191 (23.44)	1,776 (17.98)
Hypertension	7,573 (70.82)	692 (84.91)	6,881 (69.66)
Diabetes	378 (3.54)	66 (8.10)	312 (3.16)
Dyslipidemia	1,296 (12.12)	184 (22.58)	1,112 (11.26)

Values are mean±SD, n (%), median (IQR), or n/n (%).

SD indicates standard deviation; and CVD, cardiovascular disease.

Table S3. Metabolite information included in the metabolic signature

The detailed data information could be found in supplementary Excel table.

Table S2. Associations of specific healthy lifestyle factors with CVDs in the primary study

Analysis model	Healthy dietary pattern *		Never smoking status *		Normal BMI *		Regular physical activity *	
	HR (95% CI)	*p*-value	HR (95% CI)	*p*-value	HR (95% CI)	*p*-value	HR (95% CI)	*p*-value
**CVD**								
Model 1†	**0.87 (0.84, 0.90)**	<0.001	**0.61 (0.59, 0.64)**	<0.001	**0.62 (0.60, 0.64)**	<0.001	**0.87 (0.85, 0.90)**	<0.001
Model 2 ‡	**0.89 (0.86, 0.93)**	<0.001	**0.66 (0.63, 0.70)**	<0.001	**0.66 (0.63, 0.68)**	<0.001	**0.88 (0.85, 0.90)**	<0.001
Model 3 §	**0.93 (0.90, 0.97)**	<0.001	**0.70 (0.67, 0.73)**	<0.001	**0.73 (0.71, 0.76)**	<0.001	**0.91 (0.89, 0.94)**	<0.001
**IHD**								
Model 1†	**0.88 (0.84, 0.92)**	<0.001	**0.65 (0.62, 0.69)**	<0.001	**0.63 (0.61, 0.66)**	<0.001	**0.87 (0.84, 0.90)**	<0.001
Model 2 ‡	**0.91 (0.87, 0.95)**	<0.001	**0.70 (0.66, 0.74)**	<0.001	**0.66 (0.64, 0.69)**	<0.001	**0.87 (0.84, 0.91)**	<0.001
Model 3 §	**0.94 (0.90, 0.99)**	0.015	**0.74 (0.70, 0.78)**	<0.001	**0.74 (0.71, 0.77)**	<0.001	**0.91 (0.88, 0.95)**	<0.001
**Stroke**								
Model 1 †	**0.83 (0.75, 0.91)**	<0.001	**0.54 (0.48, 0.60)**	<0.001	**0.78 (0.72, 0.85)**	<0.001	0.97 (0.90, 1.04)	0.346
Model 2 ‡	**0.85 (0.77, 0.93)**	<0.001	**0.58 (0.52, 0.64)**	<0.001	**0.82 (0.75, 0.89)**	<0.001	0.97 (0.90, 1.04)	0.418
Model 3 §	**0.87 (0.79, 0.96)**	0.006	**0.60 (0.54, 0.67)**	<0.001	**0.90 (0.83, 0.98)**	0.019	1.00 (0.93, 1.08)	0.933
**MI**								
Model 1 †	**0.90 (0.81, 1.00)**	0.042	**0.48 (0.43, 0.53)**	<0.001	**0.65 (0.60, 0.71)**	<0.001	**0.85 (0.78, 0.92)**	<0.001
Model 2 ‡	0.93 (0.84, 1.03)	0.155	**0.51 (0.46, 0.57)**	<0.001	**0.69 (0.63, 0.75)**	<0.001	**0.85 (0.78, 0.92)**	<0.001
Model 3 §	0.96 (0.87, 1.07)	0.462	**0.53 (0.48, 0.60)**	<0.001	**0.76 (0.69, 0.83)**	<0.001	**0.88 (0.81, 0.95)**	0.002
**HF**								
Model 1†	**0.79 (0.73, 0.85)**	<0.001	**0.51 (0.47, 0.56)**	<0.001	**0.47 (0.44, 0.50)**	<0.001	**0.79 (0.74, 0.84)**	<0.001
Model 2 ‡	**0.82 (0.76, 0.88)**	<0.001	**0.57 (0.52, 0.62)**	<0.001	**0.50 (0.47, 0.53)**	<0.001	**0.79 (0.74, 0.84)**	<0.001
Model 3 §	**0.87 (0.81, 0.94)**	<0.001	**0.62 (0.57, 0.67)**	<0.001	**0.59 (0.55, 0.63)**	<0.001	**0.85 (0.80, 0.90)**	<0.001

* HR and 95% CI of CVDs risk per unit increment in healthy lifestyle factors.

† Adjusted for age and sex.

[‡] Based on an age- and sex-adjusted model, further adjusted for ethnicity, Townsend deprivation index, education, depression status.

[§] Based on an age- and sex-adjusted model, further adjusted for ethnicity, Townsend deprivation index, education, depression status, and metabolic signature.

CVD indicates cardiovascular disease; IHD, ischemic heart disease; MI, myocardial infarction; and HF, heart failure.

Table S3. Associations between healthy lifestyle, metabolic signature, and risk of incident CVDs in the replication study

Analysis model	Healthy lifestyle score*		Metabolic signature†	
	HR (95% CI)	*p*-value	HR (95% CI)	*p*-value
**CVD**				
Age- and sex-adjusted model	**0.87 (0.82, 0.93)**	<0.001	**0.79 (0.74, 0.85)**	<0.001
Multivariable-adjusted model‡	**0.89 (0.83, 0.95)**	<0.001	**0.80 (0.75, 0.86)**	<0.001
Multivariable-adjusted + mutual adjustment§	**0.92 (0.86, 0.99)**	0.019	**0.82 (0.76, 0.88)**	<0.001
**IHD**				
Age- and sex-adjusted model	**0.92 (0.84, 1.00)**	0.038	**0.80 (0.73, 0.87)**	<0.001
Multivariable-adjusted model‡	0.93 (0.85, 1.01)	0.075	**0.81 (0.74, 0.88)**	<0.001
Multivariable-adjusted + mutual adjustment§	0.97 (0.89, 1.05)	0.407	**0.81 (0.74, 0.89)**	<0.001
**Stroke**				
Age- and sex-adjusted model	1.04 (0.87, 1.24)	0.661	1.03 (0.86, 1.23)	0.784
Multivariable-adjusted model‡	1.06 (0.89, 1.27)	0.498	1.05 (0.87, 1.26)	0.612
Multivariable-adjusted + mutual adjustment§	1.06 (0.88, 1.26)	0.552	1.04 (0.86, 1.25)	0.699
**MI**				
Age- and sex-adjusted model	0.92 (0.76, 1.10)	0.361	**0.70 (0.57, 0.85)**	<0.001
Multivariable-adjusted model‡	0.93 (0.77, 1.12)	0.433	**0.70 (0.58, 0.86)**	<0.001
Multivariable-adjusted + mutual adjustment§	0.99 (0.82, 1.20)	0.933	**0.71 (0.58, 0.86)**	<0.001
**HF**				
Age- and sex-adjusted model	**0.68 (0.60, 0.77)**	<0.001	**0.64 (0.55, 0.74)**	<0.001
Multivariable-adjusted model‡	**0.69 (0.61, 0.79)**	<0.001	**0.65 (0.56, 0.75)**	<0.001
Multivariable-adjusted + mutual adjustment§	**0.75 (0.65, 0.85)**	<0.001	**0.70 (0.60, 0.81)**	<0.001

* HR and 95% CI of CVDs risk per SD increment in healthy lifestyle scores.

† HR and 95% CI of CVDs risk per SD increment in metabolic signature.

‡ Based on an age- and sex-adjusted model further adjusted for ethnicity, Townsend deprivation index, education, and depression status.

§ Included both healthy lifestyle score and the metabolic signature simultaneously in the multivariable-adjusted model to examine association independence.

HR indicates hazard ratio; CI, confidence interval; SD, standard deviation; CVD, cardiovascular disease; IHD, ischemic heart disease; MI, myocardial infarction; and HF, heart failure.

Table S4. Associations of metabolic signature levels with risk of incident CVDs

**Analysis model**	**Metabolic score**[*]					
	**Low**	**Moderate**		**High**		***p* for trend**
		**HR (95% CI)**	***p*-value**	**HR (95% CI)**	***p*-value**	
**CVD**						
Age- and sex-adjusted model	Ref.	**0.73 (0.70, 0.75)**	<0.001	**0.58 (0.56, 0.61)**	<0.001	<0.001
Multivariable-adjusted model[†]	Ref.	**0.75 (0.72, 0.77)**	<0.001	**0.61 (0.59, 0.64)**	<0.001	<0.001
Multivariable-adjusted + mutual adjustment[‡]	Ref.	**0.79 (0.76, 0.82)**	<0.001	**0.67 (0.65, 0.70)**	<0.001	<0.001
**IHD**						
Age- and sex-adjusted model	Ref.	**0.75 (0.72, 0.78)**	<0.001	**0.59 (0.56, 0.62)**	<0.001	<0.001
Multivariable-adjusted model[†]	Ref.	**0.77 (0.74, 0.80)**	<0.001	**0.62 (0.59, 0.65)**	<0.001	<0.001
Multivariable-adjusted + mutual adjustment[‡]	Ref.	**0.81 (0.78, 0.84)**	<0.001	**0.68 (0.65, 0.71)**	<0.001	<0.001
**Stroke**						
Age- and sex-adjusted model	Ref.	**0.75 (0.69, 0.82)**	<0.001	**0.67 (0.61, 0.74)**	<0.001	<0.001
Multivariable-adjusted model[†]	Ref.	**0.77 (0.70, 0.84)**	<0.001	**0.70 (0.64, 0.77)**	<0.001	<0.001
Multivariable-adjusted + mutual adjustment[‡]	Ref.	**0.80 (0.73, 0.87)**	<0.001	**0.75 (0.68, 0.82)**	<0.001	<0.001
**MI**						
Age- and sex-adjusted model	Ref.	**0.74 (0.67, 0.81)**	<0.001	**0.62 (0.56, 0.69)**	<0.001	<0.001
Multivariable-adjusted model[†]	Ref.	**0.76 (0.70, 0.84)**	<0.001	**0.66 (0.59, 0.74)**	<0.001	<0.001
Multivariable-adjusted + mutual adjustment[‡]	Ref.	**0.81 (0.74, 0.89)**	<0.001	**0.73 (0.66, 0.82)**	<0.001	<0.001
**HF**						
Age- and sex-adjusted model	Ref.	**0.60 (0.56, 0.64)**	<0.001	**0.45 (0.42, 0.49)**	<0.001	<0.001
Multivariable-adjusted model[†]	Ref.	**0.62 (0.58, 0.67)**	<0.001	**0.49 (0.45, 0.53)**	<0.001	<0.001
Multivariable-adjusted + mutual adjustment[‡]	Ref.	0.67 (0.63, 0.72)	<0.001	0.56 (0.51, 0.61)	<0.001	<0.001

[*] HR and 95% CI of CVDs risk per tertile in metabolic signature.

† Based on an age- and sex-adjusted model, further adjusted for ethnicity, Townsend deprivation index, education, and depression status.

‡ Based on an age- and sex-adjusted model, further adjusted for ethnicity, Townsend deprivation index, education, depression status, and healthy lifestyle.

CVD indicates cardiovascular disease; IHD, ischemic heart disease; MI, myocardial infarction; and HF, heart failure.

Table S5. Summary of 8 independent loci associated with metabolic signature

uniqID	rsID	chr	pos	A1	A2	MAF	gwasP	beta	se	nearest gene	distance	function
1:40028180:A:G	rs4660293	1	40028180	G	A	0.2346	2.28E-08	-0.17911	0.032042	PABPC4	0	intronic
1:62923863:C:T	rs12037659	1	62923863	T	C	0.3022	9.84E-55	-0.44481	0.028534	DOCK7	0	intronic
1:66058801:A:G	rs4655537	1	66058801	A	G	0.3638	1.13E-12	-0.20107	0.028263	LEPR	0	intronic
1:154428283:A:G	rs12133641	1	154428283	G	A	0.3618	1.18E-08	0.157775	0.027669	IL6R	0	intronic
1:214325218:C:T	rs1470141	1	214325218	T	C	0.2684	1.72E-08	0.175303	0.031093	PROX1	110622	intergenic
1:220970028:A:G	rs2642438	1	220970028	A	G	0.2783	3.27E-12	-0.20743	0.029776	1-Mar	0	exonic
1:234852760:C:T	rs553427	1	234852760	C	T	0.4851	4.86E-08	-0.14888	0.027285	RP4-781K5.7	0	ncRNA_intronic
10:3139264:C:T	rs111354685	10	3139264	T	C	0.3797	6.54E-09	-0.16493	0.028423	PFKP	0	intronic

Table S6. The basic characteristics of GWAS summary statistic datasets for the metabolic signature and CVDs

GWAS	Sample size	cases/controls	Number of SNPs
**Metabolic signature GWAS** (current study)	120,056	-	1,232,649
**IHD GWAS**			
CARDIoGRAM GWAS	86,995	22,233/64,762	2,420,360
**Stroke GWAS**			
MEGASTROKE consortium	446,696	40,585/406,111	8,306,090
**HF GWAS**			
HERMES Consortium	972,032	68,157/949,888	8,281,262

GWAS indicates genome-wide association studies; SNP, single nucleotide polymorphisms; CVD, cardiovascular disease; IHD, ischemic heart disease; and HF, heart failure.

Table S7. MR results for the effect of metabolic signatures on CVDs risk

Instrumental variables					Metabolic signature (current study)			Stroke (MEGASTROKE consortium)			IHD (CARDIoGRAM GWAS)			HF (HERMES Consortium)			MI (IEU Open GWAS project)		
SNP	CHR	BP	A1	A2	β	SE	*p*-value	Ln (OR)	SE	*p*-value	Ln (OR)	SE	*p*-value	Ln (OR)	SE	*p*-value	Ln (OR)	SE	*p*-value
rs11208090	1	63483437	G	A	-0.1779	0.0335	1.14E-07	-0.0088	0.0109	0.421	0.0194	0.0161	0.2282	0.0002	0.0091	0.9805	0.0002	0.0091	0.9805
rs12037659	1	62923863	T	C	-0.4448	0.0285	9.84E-55	0.0119	0.0097	0.2201	-	-	-	-0.0098	0.0083	0.2386	-0.0098	0.0083	0.2386
rs12133641	1	154428283	G	A	0.1578	0.0277	1.18E-08	0.0212	0.0093	0.0230	-	-	-	0.014	0.0081	0.08561	0.014	0.0081	0.0856
rs1470141	1	214325218	T	T	0.1753	0.0311	1.72E-08	-0.0026	0.0105	0.8087	-	-	-	-0.0087	0.0088	0.3236	-0.0087	0.0088	0.3236
rs149754280	1	213981595	A	G	0.2741	0.0528	2.15E-07	-0.0352	0.0186	0.0587	-	-	-	-0.0255	0.0221	0.2478	-0.0255	0.0221	0.2478
rs1546954	1	230303848	T	T	-0.1422	0.0279	3.56E-07	0.0135	0.0093	0.1464	-	-	-	0.016	0.008	0.0462	0.016	0.008	0.0462
rs2306714	1	207224992	A	G	-0.13791	0.0278	7.34E-07	0.006	0.0093	0.5172	0.0065	0.0142	0.6442	-0.0027	0.008	0.7333	-0.0027	0.008	0.7333
rs2642438	1	220970028	A	A	-0.2074	0.0298	3.27E-12	0.0094	0.0102	0.358	0.0197	0.0160	0.2189	-0.0011	0.0086	0.8994	-0.0011	0.0086	0.8994
rs4655537	1	66058801	A	A	-0.2011	0.0283	1.13E-12	-0.0209	0.0094	0.0266	0.0169	0.0151	0.2641	-0.0086	0.0082	0.2965	-0.0086	0.0082	0.2965
rs4660293	1	40028180	G	A	-0.1791	0.0320	2.28E-08	-0.0098	0.0109	0.3689	0.0114	0.0162	0.4807	-0.0019	0.0092	0.8358	-0.0019	0.0092	0.8358
rs4925667	1	247620654	T	T	0.1874	0.0361	2.10E-07	-0.0111	0.0125	0.3721	-	-	-	0.0016	0.0102	0.8718	0.0016	0.0102	0.8718
rs553427	1	234852760	C	C	-0.1489	0.0273	4.86E-08	-0.018	0.0092	0.0505	0.0016	0.0138	0.9099	0.0013	0.0078	0.8676	0.0013	0.0078	0.8676
rs111354685	10	3139264	T	C	-0.1649	0.0284	6.54E-09	-	-	-	-	-	-	-	-	-	-	-	-
rs6858	10	81142365	T	C	-0.1371	0.0280	9.79E-07	0.0095	0.01	0.3454	0.0375	0.0218	0.0859	-0.0085	0.0082	0.2983	-0.0085	0.0082	0.2983

GWAS indicates genome-wide association studies; MR, Mendelian randomization; CVD, cardiovascular disease; IHD, ischemic heart disease; MI, myocardial infarction; HF, heart failure; OR, odds ratio; and CI, confidence interval.

Table S8. MR sensitivity analyses investigating the effect of the metabolic signature on CVDs.

The detailed data information could be found in supplementary Excel table.

Table S9. Sensitivity analyses for associations of metabolic signature and healthy lifestyle with CVDs risk by excluding new cases within 2 years

**Analysis model**	**Healthy lifestyle score***		**Metabolic signature†**	
	**HR (95% CI)**	***p*-value**	**HR (95% CI)**	***p*-value**
**CVD**				
Age- and sex-adjusted model	**0.79 (0.78, 0.81)**	<0.001	**0.78 (0.76, 0.79)**	<0.001
Multivariable-adjusted model‡	**0.82 (0.80, 0.83)**	<0.001	**0.79 (0.78, 0.81)**	<0.001
Multivariable-adjusted + mutual adjustment§	**0.85 (0.84, 0.86)**	<0.001	**0.83 (0.81, 0.84)**	<0.001
**IHD**				
Age- and sex-adjusted model	**0.80 (0.79, 0.81)**	<0.001	**0.78 (0.77, 0.79)**	<0.001
Multivariable-adjusted model‡	**0.82 (0.81, 0.84)**	<0.001	**0.80 (0.78, 0.81)**	<0.001
Multivariable-adjusted + mutual adjustment§	**0.86 (0.84, 0.87)**	<0.001	**0.83 (0.81, 0.85)**	<0.001
**Stroke**				
Age- and sex-adjusted model	**0.85 (0.81, 0.88)**	<0.001	**0.83 (0.80, 0.87)**	<0.001
Multivariable-adjusted model‡	**0.86 (0.83, 0.90)**	<0.001	**0.85 (0.81, 0.88)**	<0.001
Multivariable-adjusted + mutual adjustment§	**0.89 (0.86, 0.93)**	<0.001	**0.87 (0.84, 0.91)**	<0.001
**MI**				
Age- and sex-adjusted model	**0.79 (0.76, 0.82)**	<0.001	**0.81 (0.77, 0.84)**	<0.001
Multivariable-adjusted model‡	**0.82 (0.78, 0.85)**	<0.001	**0.83 (0.79, 0.87)**	<0.001
Multivariable-adjusted + mutual adjustment§	**0.84 (0.81, 0.88)**	<0.001	**0.87 (0.83, 0.91)**	<0.001
**HF**				
Age- and sex-adjusted model	**0.70 (0.68, 0.72)**	<0.001	**0.68 (0.66, 0.70)**	<0.001
Multivariable-adjusted model‡	**0.73 (0.71, 0.75)**	<0.001	**0.70 (0.68, 0.73)**	<0.001
Multivariable-adjusted + mutual adjustment§	**0.78 (0.76, 0.80)**	<0.001	**0.75 (0.72, 0.77)**	<0.001

* HR and 95% CI of CVDs risk per SD increment in healthy lifestyle scores.

† HR and 95% CI of CVDs risk per SD increment in metabolic signature.

‡ Based on an age- and sex-adjusted model further adjusted for ethnicity, Townsend deprivation index, education, and depression status.

§ Included both healthy lifestyle score and the metabolic signature simultaneously in the multivariable-adjusted model to examine association independence.

HR indicates hazard ratio; CI, confidence interval; SD, standard deviation; CVD, cardiovascular disease; IHD, ischemic heart disease; MI, myocardial infarction; and HF, heart failure.

Table S10. Sensitivity analyses for associations of metabolic signature and healthy lifestyle with CVDs risk adjustment for prevalent cancer diseases

Analysis model	Healthy lifestyle score*		Metabolic signature†	
	HR (95% CI)	*p*-value	HR (95% CI)	*p*-value
**CVD**				
Multivariable-adjusted model‡	**0.81 (0.80, 0.83)**	<0.001	**0.79 (0.78, 0.81)**	<0.001
Multivariable-adjusted + mutual adjustment§	**0.85 (0.83, 0.86)**	<0.001	**0.83 (0.81, 0.84)**	<0.001
**IHD**				
Multivariable-adjusted model‡	**0.82 (0.80, 0.83)**	<0.001	**0.80 (0.78, 0.81)**	<0.001
Multivariable-adjusted + mutual adjustment§	**0.85 (0.84, 0.87)**	<0.001	**0.83 (0.81, 0.85)**	<0.001
**Stroke**				
Multivariable-adjusted model‡	**0.86 (0.83, 0.90)**	<0.001	**0.84 (0.80, 0.87)**	<0.001
Multivariable-adjusted + mutual adjustment§	**0.89 (0.86, 0.93)**	<0.001	**0.86 (0.83, 0.90)**	<0.001
**MI**				
Multivariable-adjusted model‡	**0.80 (0.77, 0.83)**	<0.001	**0.82 (0.78, 0.85)**	<0.001
Multivariable-adjusted + mutual adjustment§	**0.83 (0.79, 0.86)**	<0.001	**0.86 (0.82, 0.90)**	<0.001
**HF**				
Multivariable-adjusted model‡	**0.73 (0.71, 0.75)**	<0.001	**0.70 (0.68, 0.72)**	<0.001
Multivariable-adjusted + mutual adjustment§	**0.78 (0.75, 0.80)**	<0.001	**0.74 (0.72, 0.77)**	<0.001

* HR and 95% CI of CVDs risk per SD increment in healthy lifestyle scores.

† HR and 95% CI of CVDs risk per SD increment in metabolic signature.

‡ Based on an age- and sex-adjusted model further adjusted for ethnicity, Townsend deprivation index, education, depression status, and cancer cases.

§ Included both healthy lifestyle score and the metabolic signature simultaneously in the multivariable-adjusted model to examine association independence.

HR indicates hazard ratio; CI, confidence interval; SD, standard deviation; CVD, cardiovascular disease; IHD, ischemic heart disease; MI, myocardial infarction; and HF, heart failure.

Table S11. Sensitivity analyses for associations of metabolic signature and healthy lifestyle with CVDs risk adjusted for traditional risk factors

Analysis model	Healthy lifestyle score*		Metabolic signature†	
	HR (95% CI)	*p*-value	HR (95% CI)	*p*-value
**CVD**				
Multivariable-adjusted model‡	**0.87 (0.85, 0.88)**	<0.001	**0.87 (0.85, 0.88)**	<0.001
Multivariable-adjusted + mutual adjustment§	**0.88 (0.87, 0.90)**	<0.001	**0.88 (0.86, 0.90)**	<0.001
**IHD**				
Multivariable-adjusted model‡	**0.87 (0.86, 0.89)**	<0.001	**0.88 (0.86, 0.90)**	<0.001
Multivariable-adjusted + mutual adjustment§	**0.88 (0.87, 0.90)**	<0.001	**0.89 (0.88, 0.91)**	<0.001
**Stroke**				
Multivariable-adjusted model‡	**0.87 (0.84, 0.91)**	<0.001	**0.87 (0.83, 0.91)**	<0.001
Multivariable-adjusted + mutual adjustment§	**0.89 (0.85, 0.92)**	<0.001	**0.88 (0.85, 0.92)**	<0.001
**MI**				
Multivariable-adjusted model‡	**0.85 (0.81, 0.89)**	<0.001	**0.88 (0.84, 0.92)**	<0.001
Multivariable-adjusted + mutual adjustment§	**0.86 (0.82, 0.90)**	<0.001	**0.89 (0.85, 0.94)**	<0.001
**HF**				
Multivariable-adjusted model‡	**0.82 (0.80, 0.85)**	<0.001	**0.79 (0.77, 0.82)**	<0.001
Multivariable-adjusted + mutual adjustment§	**0.84 (0.81, 0.87)**	<0.001	**0.81 (0.78, 0.84)**	<0.001

* HR and 95% CI of CVDs risk per SD increment in healthy lifestyle scores.

† HR and 95% CI of CVDs risk per SD increment in metabolic signature.

‡ Based on an age- and sex-adjusted model further adjusted for ethnicity, Townsend deprivation index, education, depression status, and traditional risk factors.

§ Included both healthy lifestyle score and the metabolic signature simultaneously in the multivariable-adjusted model to examine association independence.

HR indicates hazard ratio; CI, confidence interval; SD, standard deviation; CVD, cardiovascular disease; IHD, ischemic heart disease; MI, myocardial infarction; and HF, heart failure.

Table S12. Sensitivity analyses for associations of metabolic signature and healthy lifestyle with CVDs risk adjusted for medications

Analysis model	Healthy lifestyle score*		Metabolic signature†	
	HR (95% CI)	*p*-value	HR (95% CI)	*p*-value
**CVD**				
Multivariable-adjusted model‡	**0.84 (0.83, 0.85)**	<0.001	**0.85 (0.83, 0.86)**	<0.001
Multivariable-adjusted + mutual adjustment§	**0.86 (0.85, 0.87)**	<0.001	**0.88 (0.86, 0.89)**	<0.001
**IHD**				
Multivariable-adjusted model‡	**0.85 (0.83, 0.86)**	<0.001	**0.86 (0.85, 0.88)**	<0.001
Multivariable-adjusted + mutual adjustment§	**0.87 (0.85, 0.88)**	<0.001	**0.89 (0.88, 0.91)**	<0.001
**Stroke**				
Multivariable-adjusted model‡	**0.88 (0.85, 0.91)**	<0.001	**0.86 (0.83, 0.90)**	<0.001
Multivariable-adjusted + mutual adjustment§	**0.90 (0.86, 0.93)**	<0.001	**0.89 (0.85, 0.92)**	<0.001
**MI**				
Multivariable-adjusted model‡	**0.83 (0.80, 0.86)**	<0.001	**0.85 (0.82, 0.89)**	<0.001
Multivariable-adjusted + mutual adjustment§	**0.85 (0.81, 0.88)**	<0.001	**0.89 (0.85, 0.93)**	<0.001
**HF**				
Multivariable-adjusted model‡	**0.75 (0.73, 0.77)**	<0.001	**0.75 (0.73, 0.78)**	<0.001
Multivariable-adjusted + mutual adjustment§	**0.79 (0.76, 0.81)**	<0.001	**0.80 (0.77, 0.82)**	<0.001

* HR and 95% CI of CVDs risk per SD increment in healthy lifestyle scores.

† HR and 95% CI of CVDs risk per SD increment in metabolic signature.

‡ Based on an age- and sex-adjusted model further adjusted for ethnicity, Townsend deprivation index, education, medications, and depression status.

§ Included both healthy lifestyle score and the metabolic signature simultaneously in the multivariable-adjusted model to examine association independence.

HR indicates hazard ratio; CI, confidence interval; SD, standard deviation; CVD, cardiovascular disease; IHD, ischemic heart disease; MI, myocardial infarction; and HF, heart failure.

Table S13. Sensitivity analyses for associations of metabolic signature and healthy lifestyle with CVDs risk with imputation of incomplete lifestyle factors

Analysis model	Healthy lifestyle score*		Metabolic signature†	
	HR (95% CI)	*p*-value	HR (95% CI)	*p*-value
**CVD**				
Age- and sex-adjusted model	**0.85 (0.84, 0.86)**	<0.001	**0.88 (0.87, 0.89)**	<0.001
Multivariable-adjusted model‡	**0.87 (0.86, 0.88)**	<0.001	**0.89 (0.88, 0.90)**	<0.001
Multivariable-adjusted + mutual adjustment§	**0.88 (0.86, 0.89)**	<0.001	**0.90 (0.88, 0.91)**	<0.001
**IHD**				
Age- and sex-adjusted model	**0.86 (0.85, 0.87)**	<0.001	**0.89 (0.88, 0.91)**	<0.001
Multivariable-adjusted model‡	**0.87 (0.86, 0.89)**	<0.001	**0.90 (0.89, 0.91)**	<0.001
Multivariable-adjusted + mutual adjustment§	**0.88 (0.87, 0.89)**	<0.001	**0.91 (0.90, 0.92)**	<0.001
**Stroke**				
Age- and sex-adjusted model	**0.89 (0.87, 0.92)**	<0.001	**0.86 (0.83, 0.89)**	<0.001
Multivariable-adjusted model‡	**0.91 (0.88, 0.94)**	<0.001	**0.87 (0.84, 0.89)**	<0.001
Multivariable-adjusted + mutual adjustment§	**0.92 (0.89, 0.95)**	<0.001	**0.87 (0.85, 0.90)**	<0.001
**MI**				
Age- and sex-adjusted model	**0.84 (0.81, 0.87)**	<0.001	**0.84 (0.81, 0.87)**	<0.001
Multivariable-adjusted model‡	**0.86 (0.83, 0.88)**	<0.001	**0.85 (0.82, 0.88)**	<0.001
Multivariable-adjusted + mutual adjustment§	**0.87 (0.84, 0.90)**	<0.001	**0.86 (0.83, 0.89)**	<0.001
**HF**				
Age- and sex-adjusted model	**0.77 (0.75, 0.79)**	<0.001	**0.80 (0.78, 0.82)**	<0.001
Multivariable-adjusted model‡	**0.79 (0.77, 0.81)**	<0.001	**0.81 (0.79, 0.83)**	<0.001
Multivariable-adjusted + mutual adjustment§	**0.80 (0.79, 0.82)**	<0.001	**0.83 (0.81, 0.85)**	<0.001

* HR and 95% CI of CVDs risk per SD increment in healthy lifestyle scores.

† HR and 95% CI of CVDs risk per SD increment in metabolic signature.

‡ Based on an age- and sex-adjusted model further adjusted for ethnicity, Townsend deprivation index, education, and depression status.

§ Included both healthy lifestyle score and the metabolic signature simultaneously in the multivariable-adjusted model to examine association independence.

HR indicates hazard ratio; CI, confidence interval; SD, standard deviation; CVD, cardiovascular disease; IHD, ischemic heart disease; MI, myocardial infarction; and HF, heart failure.

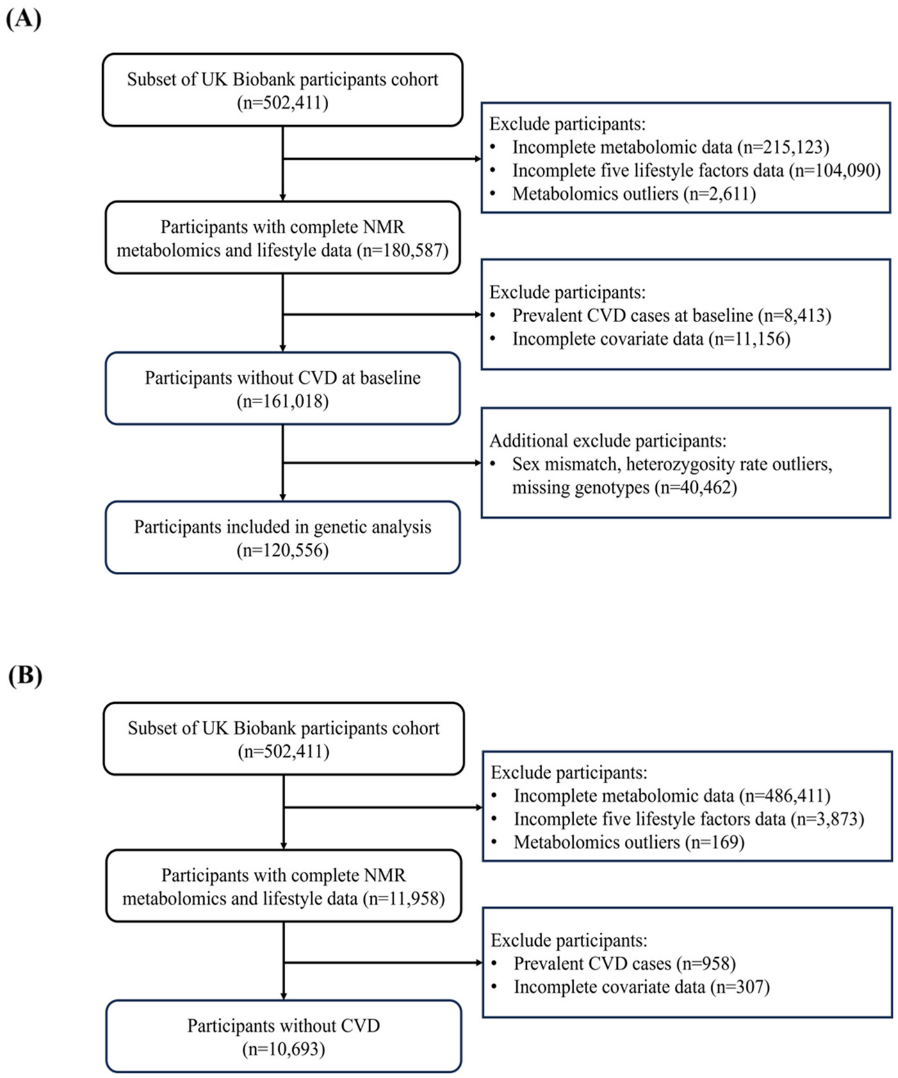

Figure S1. Flowchart of study participant selection. (A) primary study. (B) replication study. NMR indicates nuclear magnetic resonance; and CVD, cardiovascular disease.

The detailed information were included in PDF.

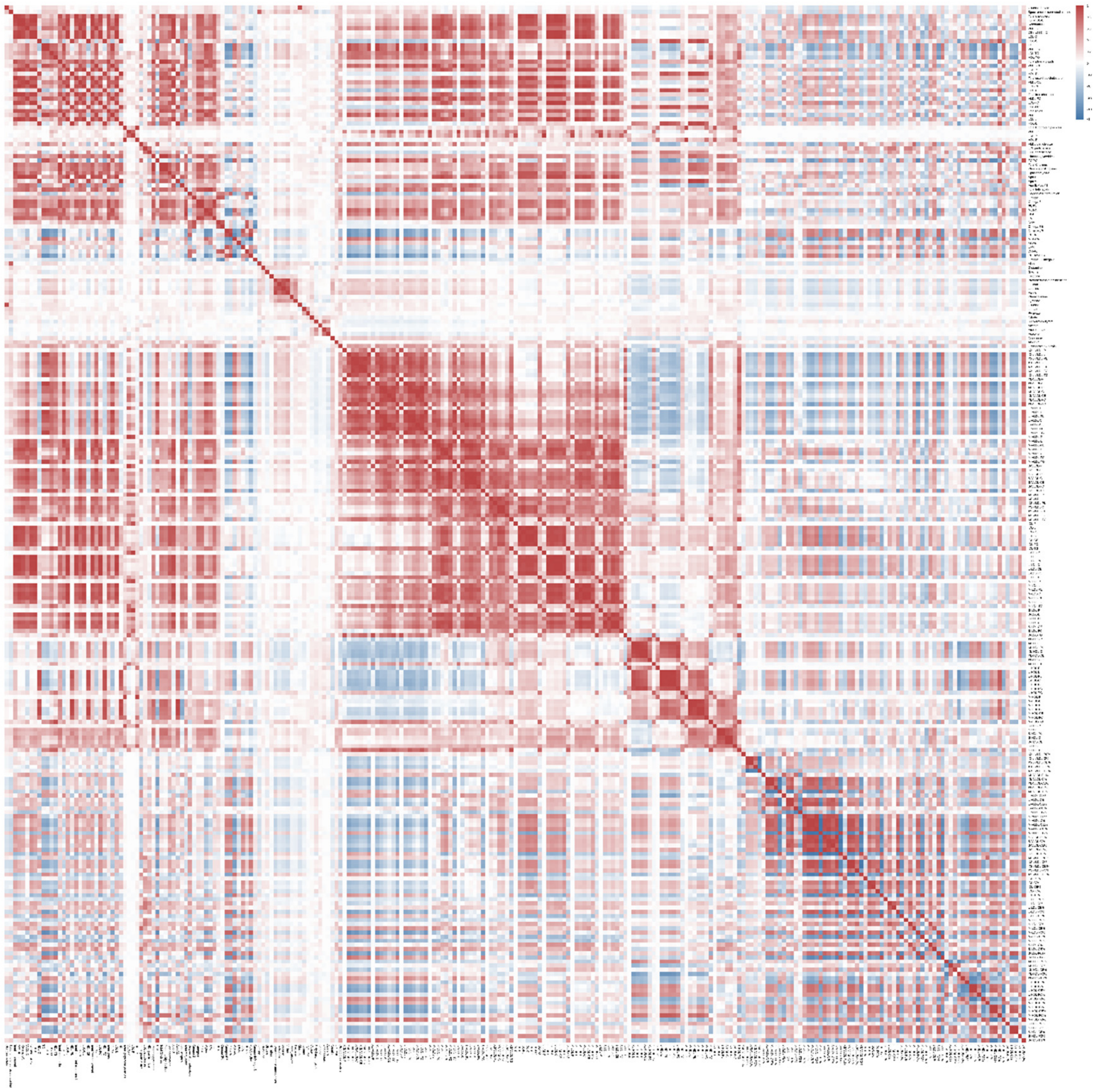

Figure S2. Correlation matrix for the 251 metabolites.

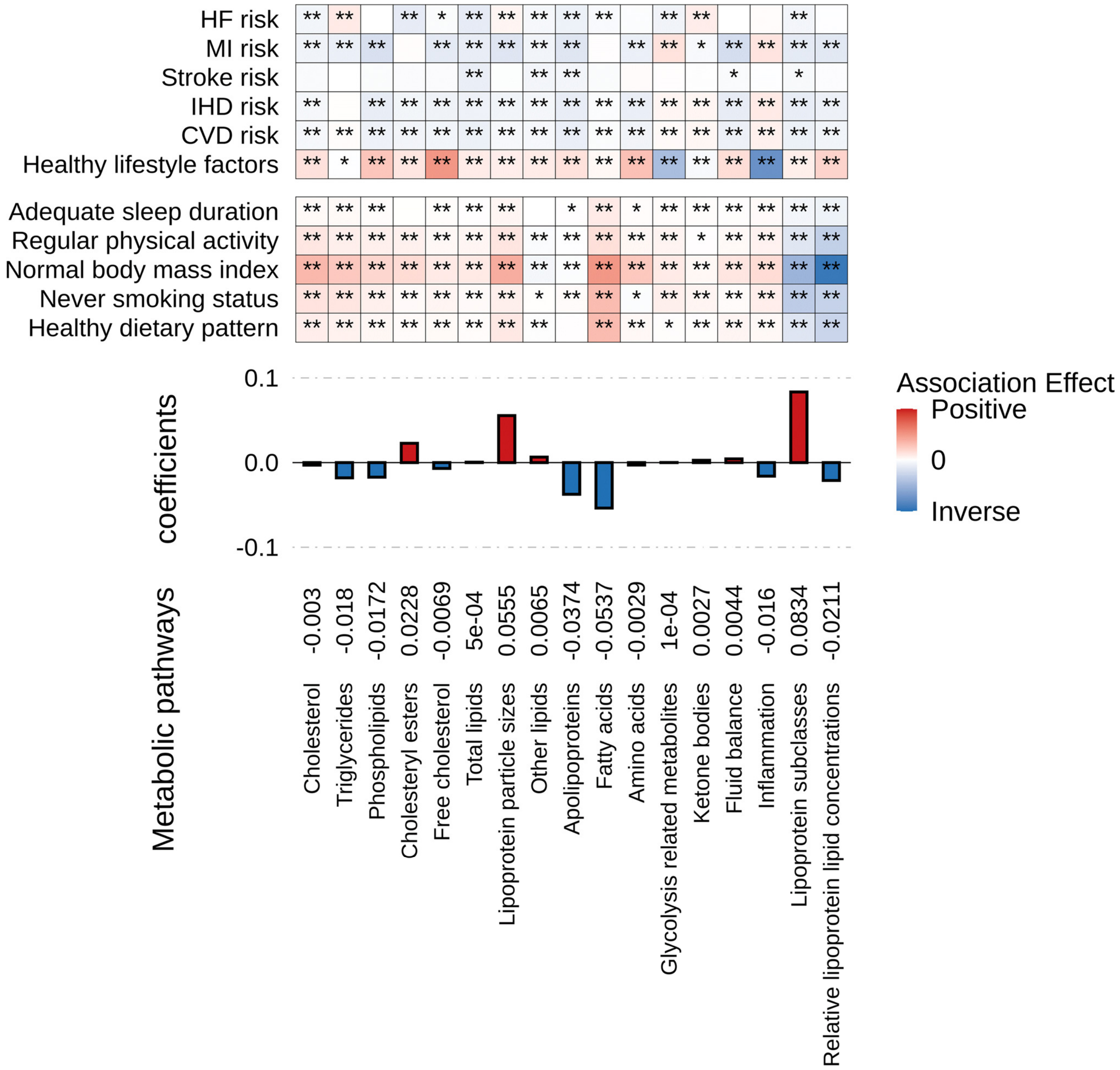

Figure S3. Associations of the 17 metabolic pathways constituting the metabolic signature and healthy lifestyle components, healthy lifestyle score, and subsequent CVDs. From bottom to top are the metabolic pathway's coefficients (weights) of the signature and associations with healthy lifestyle component, healthy lifestyle, and CVDs. Coefficients for healthy lifestyle components and healthy lifestyle score are for one unit increment per healthy lifestyle component's score. Coefficients for CVD risk are for per SD increment in metabolic pathways. Colors indicate the association directions (positive-red and inverse-blue) and magnitudes (the darker the color, the bigger the magnitude); asterisks represent significance levels (**p*< 0.05 and ** *p*< Bonferroni corrected 0.05. We used Bonferroni-corrected for 17 metabolic pathways for the healthy lifestyle score and CVD risk and 17 pathways x 5 healthy lifestyle components). CVD indicates cardiovascular disease; IHD, ischemic

heart disease; MI, myocardial infarction; and HF, heart failure.

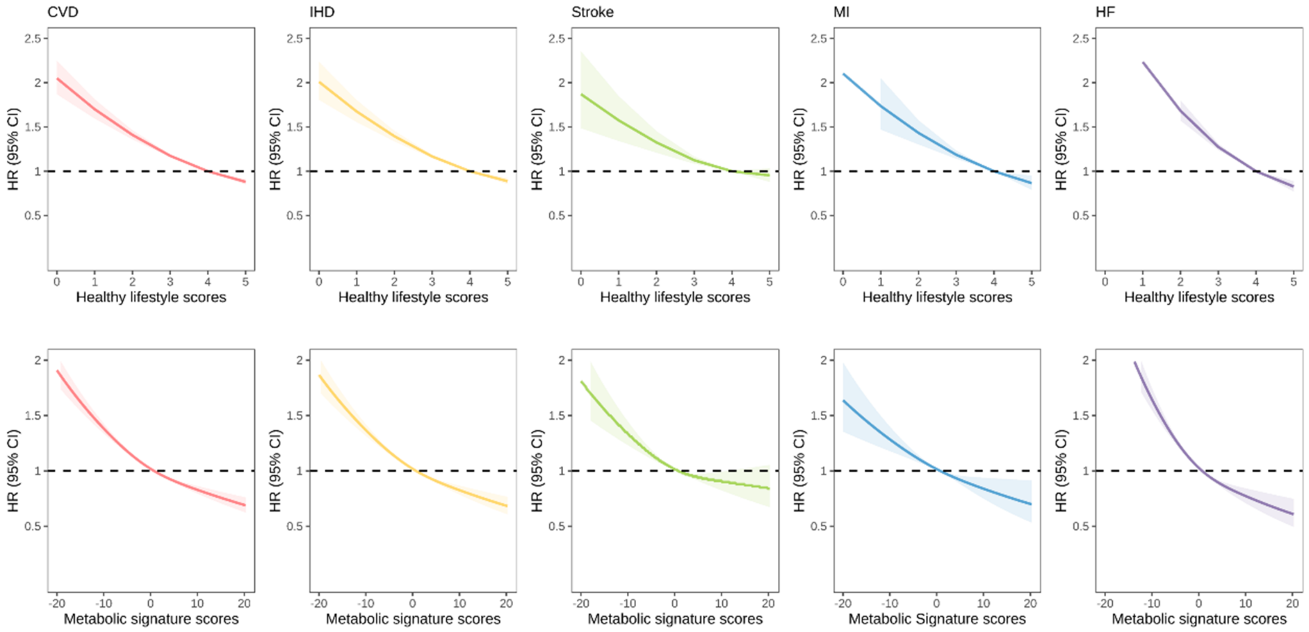

Figure S4. Dose-response relationship between healthy lifestyle, metabolic signature, and CVDs risk using restricted cubic spline analysis.

Adjusted for age, sex, ethnicity, Townsend deprivation index, education, and depression status. HR indicates hazard ratio; CI, confidence interval; CVD, cardiovascular disease; IHD, ischemic heart disease; MI, myocardial infarction; and HF, heart failure.

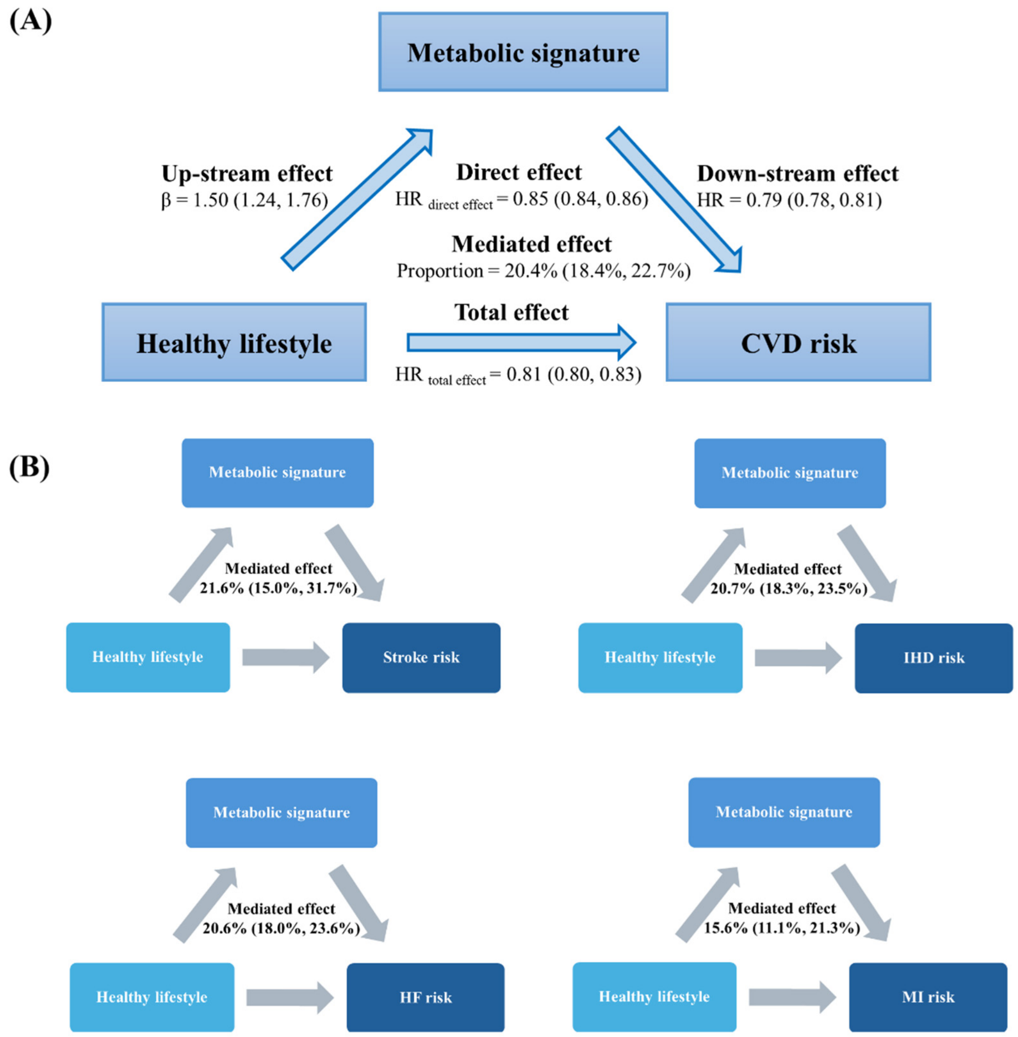

Figure S5. Mediation analyses of the association of healthy lifestyle with different diseases by the metabolic signature. (A) for CVD. (B) for stroke, IHD, HF, and MI. Adjusted for age, sex, ethnicity, Townsend deprivation index, education, and depression status. HR indicates hazard ratio; CI, confidence interval; CVD, cardiovascular disease; IHD, ischemic heart disease; MI, myocardial infarction; and HF, heart failure.

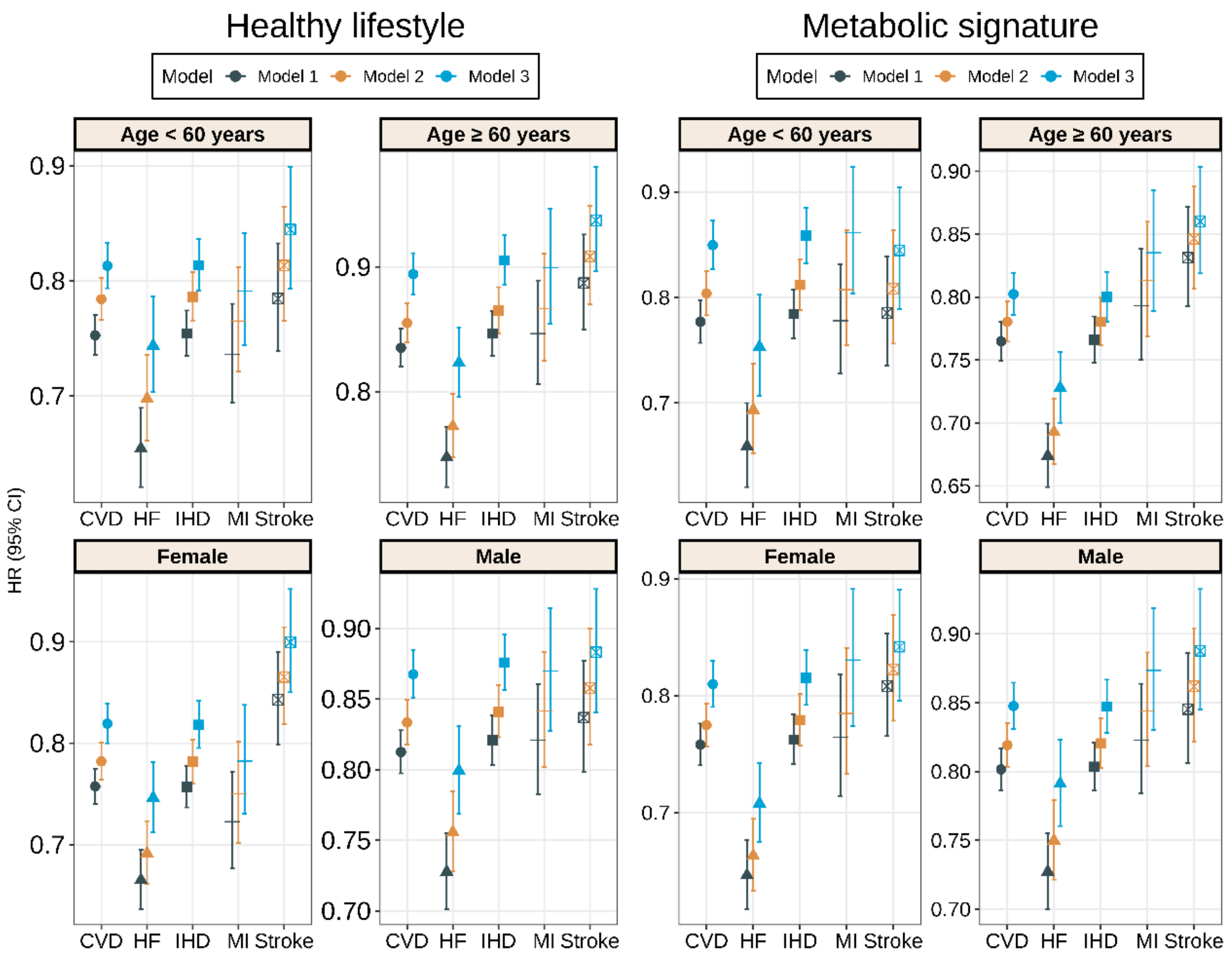

Figure S6. Sensitivity analyses for associations of metabolic signature and healthy lifestyle with CVDs risk stratified by age and sex.

Adjusted for age, sex, ethnicity, Townsend deprivation index, education, and depression status. HR indicates hazard ratio; CI, confidence interval; CVD, cardiovascular disease; IHD, ischemic heart disease; MI, myocardial infarction; and HF, heart failure.
